# Solid-State Arrangement and Magnetic Properties of Hybrid Copper(II)-L-Arginine 1D Coordination Polymer Based on Nitrate Bridges

**DOI:** 10.1021/acs.cgd.6c00298

**Published:** 2026-07-10

**Authors:** Agnieszka Wojciechowska, Waldemar Maniukiewicz, Lesław Sieroń, Saied M. Soliman, Maria Jerzykiewicz, Maria Korabik, Ivo Heinmaa, Joosep Link, Raivo Stern, Jan Novotný, Radek Marek

**Affiliations:** † Faculty of Chemistry, Wroclaw University of Science and Technology, Wyb. Wyspiańskiego 27, 50−370 Wroclaw, Poland; ‡ Institute of General and Ecological Chemistry, Łódź University of Technology, Żeromskiego 116, 90−924 Łódź, Poland; § Department of Chemistry, 110113 Faculty of Science Alexandria University, P.O. Box 426, Ibrahimia, Alexandria 8, 21321, Egypt; ∥ Faculty of Chemistry, University of Wrocław, Joliot−Curie 14, 50-383 Wrocław, Poland; ⊥ Department of Chemical Physics, National Institute of Chemical Physics and Biophysics, Akadeemia tee 23, EE−12618 Tallinn, Estonia; #CEITEC–Central European Institute of Technology and ∇National Center for Biomolecular Research, Faculty of Science, Masaryk University, CZ−62500 Brno, Czechia

## Abstract

Copper­(II) coordination compounds are prospective building blocks of magnetic materials for future technologies and biologically active compounds. Their magnetic properties are greatly influenced by coordinating ligands, counterions, cocrystallization partners, and crystal arrangements. The self-organization of crystal structures containing [Cu­(Arg)­(B)]^2+^ cationic complexes is largely determined by the type of counteranions. Using NO_3_^–^ ions resulted in the formation of a new copper­(II) l–arginato 1D polymeric complex, with the following formula: {[Cu­( l–Arg)­(phen)­(*μ*-NO_3_)]­(NO_3_)·H_2_O}_n_. The results demonstrate the importance of interactions between NO_3_^–^ and the [Cu­(l–Arg)­(phen)]^2+^ coordination units in constructing the unit cell, stabilizing the structure and the spectroscopic and magnetic properties. The [Cu­(l–Arg)­(phen)]^2+^ cations are linked by NO_3_^–^ axial–axial bridges with Cu···Cu distance of 7.071 Å, which induced weak antiferromagnetic interactions between copper­(II) ions with S = ^1^/_2_. The O···H intermolecular interactions mostly control the molecular packing. The EPR (*g*_⊥_ = 2.056, *g*_||_ = 2.230) and vis (16970 cm^–1^) parameters confirm the elongated octahedral geometry (*T* = 0.76) and SOMO oriented in the *xy* plane N_3_O coordination sphere. Solid-state ^13^C NMR spectroscopy is used to characterize the distribution of spin density in organic ligands, with relativistic two-component (SO-ZORA) DFT calculations used to interpret experimental ^13^C NMR data. Our current N_3_O coordination polymer belongs to an interesting class of weakly coupled antiferromagnetic compounds.

## Graphical abstract

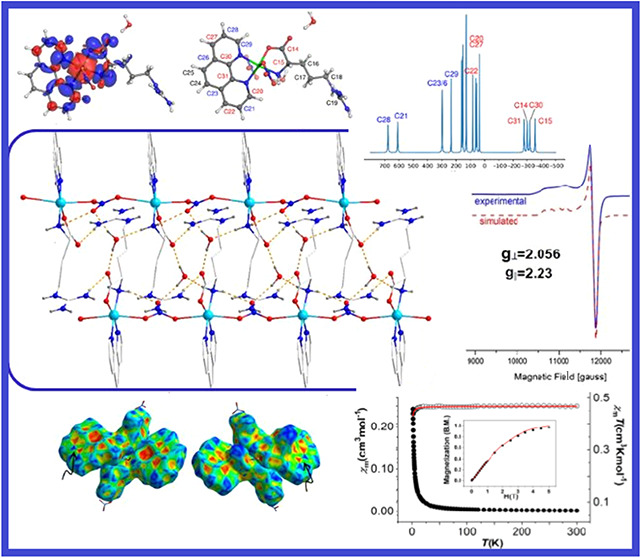

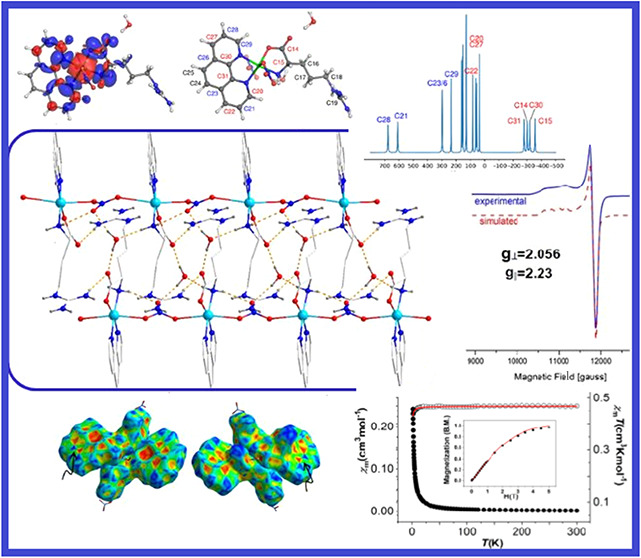

## 1 Introduction

Hybrid organic–inorganic coordination polymers (CPs) exhibit a wide range of structures, from one-dimensional (1D) chains to three-dimensional (3D) frameworks. This structural diversity allows for their different functions and applications in gas adsorption, luminescence, catalysis, magnetism, nonlinear optics, and medicine.^1–7^ The most feasible strategy for designing and constructing coordination polymers is an appropriate selection of suitable organic and inorganic ligands that enable control over the structural motifs. For this reason, in the construction of CPs, the (*S*)-2-amino-5-guanidinopentanoic acid ( l–arginine = l–Arg) is used, which involves the carboxylate O-donors as linkers in the chemistry of homo- and heterometallic CPs.^8–12^ However, l–Arg zwitterion primarily functions as a chelating ligand, involving the donor atoms N_NH_2__ and O_COO_^–^, and forms stable complexes of general formulas [M­(Arg)_2_]^2+^ or [M­(Arg)­(B)]^2+^ (where M = Cu­(II), Zn­(II), Ag­(I), Co­(II), Pt­(II); B = organic co-ligands).^13–40^ The charge of coordination cations, [M­(Arg)_2_]^2+^ and [M­(Arg)­(B)]^2+^, must be balanced by the various counteranions such as SO_4_^2–^ ^22^ or aromatic carboxylates.^21^ ,^22^ ,^26^ These anions are key factors in determining (i) *cis*–*trans* complex isomerism and (ii) self-organization, particularly in structures containing [Cu­(Arg)_2_]^2+^ complex cations, resulting in a layered structure, single-helical structure, and double-helical structure. Some inorganic anions, such as hetero polymolybdates,^13^ nitrates,^14,19,10,23,35^ oxalates,^25^ or perchlorates,^20^ have also been applied as bridges during the design of l–arginato coordination polymers.

Our research has also focused on l–argininato copper­(II) complexes, mainly investigating the properties of monomeric and polymeric [M­(l–Arg)_2_(X)]Y compounds (M = Cu­(II), Zn­(II); X = H_2_O, NCS^–^, 4,4′–bpy; Y = C_2_O_4_^2–^, NCS^–^, Cl^–^).^17,25,33,37^ Our recent work has been focused on the structural organization and properties of crystals containing ternary [Cu­(Arg)­(B)]^2+^ cation complexes with the heterocyclic base (B = phen, bpy) balanced by Cl^–^, Br^–^, or NO_3_^–^ ions. In the crystal structure of [CuCl­(l–Arg)­(phen)]­Cl·2H_2_O and [CuCl­(l–Arg)­(bpy)]­Cl·3H_2_O, the [CuCl­(l–Arg)­(B)]^+^ units are linked in dimeric units, which form 1D *zig–zag* chains based on hydrogen bonds.^32,35^ The presence of Br^–^ ions in [CuBr­(l–Arg)­(bpy)]­Br·2H_2_O has resulted in a ribbon-like organization.^36^ In contrast, the mononuclear [Cu­(l-Arg)­(bpy)]^2+^ units are joined by the coordinated nitrate ions to form infinite 1D coordination chains in the structure of {[Cu­(l–Arg)­(bpy)­(*μ*–NO_3_)]­NO_3_}_n_.^35^

Continuing the study of the influence of inorganic anions on the crystal structure involving [Cu­(Arg)­(B)]^2+^ entities and their magnetic properties, in this work, we have investigated the structural organization and magnetic properties of a new copper­(II) arg system of formula {[Cu­(l–Arg)­(phen)­(*μ*–NO_3_)]­(NO_3_)·H_2_O}_n_ (**1**). The complex shows the 1D polymeric nature due to the presence of linked ternary [Cu­(l–Arg)­(phen)]^2+^ units, which are held together by NO_3_^–^ ions. The structural characterization of **1** has been complemented by theoretical Hirshfeld surface analysis. In parallel, the structural self-organization and spectroscopic properties of compound **1** have been carefully compared with the structurally related {[Cu­(l–Arg)­(bpy)­(*μ*–NO_3_)]­(NO_3_)}_n_ compound.^35^ A range of spectroscopic techniques, including EPR and pNMR, has been used to characterize the distribution of spin density in the system with support by two-component relativistic DFT calculations of EPR and NMR parameters.

## 2 Experimental Section

### 2.1 Materials

Phen monohydrate was obtained from Polish Chemical Reagents. Cu­(NO_3_)_2_·2.5H_2_O and l–arg were purchased from Sigma. All substrates were used as received. They were not further purified.

### 2.2 Synthesis of Compound **1**

10 mL of water solution containing 0.5 mmol of l–arg (87.1 mg) was added dropwise, with stirring, to 10 mL of an aqueous solution of copper­(II) nitrate hemi­(pentahydrate) (0.5 mmol, 116.3 mg). After 10 min of continuous stirring, a methanol solution of phen monohydrate (0.5 mmol, 99.1 mg, 10 mL) was added dropwise. The resulting solution, which was a deep blue color, was left to evaporate at room temperature. After 1 month, the blue crystals of compound **1** were collected, filtered, and air-dried. *Anal*. C_18_H_24_CuN_8_O_9_ *Calcd* (%): C, 38.60; H, 4.29; N, 20.01. Found: C, 38.25; H, 4.13; N, 19.20. An Elmer Vario EL III Analyzer was used for elemental analyses of CHN content in complex.

### 2.3 Powder X-ray Diffraction (PXRD) and Single-Crystal X-ray Diffraction

The phase purity of compound **1** was verified by powder X-ray diffraction measurements. PXRD data (Figure S1a, Supporting Information) were collected using a PANalytical X’Pert Pro MPD diffractometer (Malvern Panalytical Ltd., Royston, UK) operating in Bragg–Brentano reflection geometry with Cu Kα radiation generated from a sealed X-ray tube. Diffraction patterns were recorded over the 2θ range of 5–55° using a step size of 0.0167° and a counting time of 20 s per step. To reduce the influence of preferred orientation, the sample was continuously rotated during the measurement. The experimental powder diffraction pattern was subsequently compared with the pattern simulated from single-crystal structural data (Figure S1b). The close correspondence between the two data sets confirmed the phase purity and homogeneity of the synthesized material.

Single-crystal X-ray diffraction data were collected at 100 K using a RIGAKU XtaLAB Synergy diffractometer equipped with a Dualflex goniometer, a Pilatus 300 K detector, and a PhotonJet microfocus X-ray source. Measurements were carried out using Cu Kα radiation (λ = 1.54184 Å) and the ω-scan acquisition mode. Data collection, unit-cell refinement, data reduction, and absorption corrections were performed with the CrysAlis PRO software package.^41^ The crystal structure was solved by direct methods employing the SHELXT 2018/3 program.^42^ Atomic scattering factors were taken from the International Tables for X-ray Crystallography. The structural model was refined against F^2^ by full-matrix least-squares procedures using SHELXL 2018/3.^43^ All nonhydrogen atoms were refined anisotropically. The hydrogen atom involved in hydrogen bonding was located from a Fourier difference map and refined without constraints, whereas all remaining hydrogen atoms were positioned geometrically (C–H = 0.93–0.98 Å) and treated using a riding model. Their isotropic displacement parameters were fixed at 1.2U_eq_ of the corresponding parent atoms. Detailed crystallographic parameters and refinement statistics are summarized in Table S1. Hirshfeld surface and topological analyses were carried out using Crystal Explorer 17.5 software.^44^

### 2.4 Spectroscopic Methods (FT–IR/Raman/FIR, NIR–Vis–UV, X- and Q-Band EPR)

ATR FT–IR spectra of **1** and the ligands (l–Arg and phen) were measured on a Bruker Vertex 70 (Bruker Optics, Ettlingen, Germany) Fourier transform Infrared spectrometer, which was equipped with an ATR accessory and an air-cooled DTGS detector. Spectra were acquired at room temperature, with a resolution of 2 cm^–1^ and with 64 scans in the IR (4000–400 cm^–1^) and FIR (600–100 cm^–1^) regions (both background and sample spectra were acquired with 64 scans per measurement). The OPUS Software (ver. 7.5, Bruker Optics, Ettlingen, Germany) was used. The Raman spectrum of **1** was recorded with a dispersive Senterra II spectrometer (Bruker), coupled to a confocal microscope with lenses of different focal lengths. The presented spectrum was recorded for **1** in the range of 4450–45 cm^–1^ with a resolution of 9–18 cm^–1^, using a 532 nm laser excitation line and a 25 μm × 1000 μm aperture. The Raman spectra of the ligands were measured on a Bruker MultiRAM spectrometer (Nd:YAG laser, 1064 nm) with a resolution of 2 cm^–1^.

The diffuse-reflectance electronic spectra of **1** and the ligands in the NIR–UV/vis range were collected on a Cary 500 Scan spectrophotometer. Measurements were taken at 10 cm^–1^ intervals within the range of 5000–50,000 cm^–1^ at room temperature, using the same parameters as the white reference sample.

EPR spectra were measured using a Bruker Elexsys E 500 Spectrometer equipped with an NMR tesla meter (ER 036TM) and Bruker frequency counters. The Li/LiH standard was used for tesla meter calibration, resulting in the measurements’ precision of *g* parameter 0.0001 and A·10^–4^ cm^–1^. X- and Q-band measurements were performed for powdered samples at 293 and 77 K (X-band) and at 293 K (Q-band). Measurement parameters for the X-band and Q-band EPR were as follows: microwave power 10 mW and modulation amplitude 10 G. The *g* parameters and hyperfine parameters (A_∥_ and A_⊥_) were determined using SimFonia 1.26, which is distributed by Bruker.

### 2.5 Magnetic Properties

The magnetic properties of a powder sample of the compound (21.35 mg) were investigated using a Quantum Design SQUID Magnetometer (type MPMS-XL5). The calculations took into account the diamagnetism of the constituent atoms (Pascal constants) and the temperature-independent paramagnetism of the copper­(II) ion (60·10^–6^ cm^3^ mol^–1^).^45^ The expression $\mu_{\mathrm{eff}}=2.83\sqrt{\chi_{m}T}$ ­(B.M.) was used to calculate magnetic moments.

In the fitting procedure of exchange parameters, a good agreement factor was defined as Eq. (1):

1 $$R=\Sigma_{i=1}^{n}\frac{{\left(\chi_{i}^{\exp}T-\chi_{i}^{\mathrm{calc}}T\right)}^{2}}{{\left(\chi_{i}^{\exp}T\right)}^{2}}$$

### 2.6 ^13^C MAS NMR Spectroscopy and DFT Calculation of Paramagnetic NMR Shifts

#### 2.6.1 NMR

^13^C MAS spectra were recorded on a Bruker AVANCE-II spectrometer at a 4.7 T magnetic field using a home-built MAS probe for 1,8 mm × 15 mm Si_3_N_4_ rotors. The spinning speed of the sample was 35 kHz. The spectrum was recorded with an ordinary spin echo pulse sequence π/2−τ–π–τ *acquisition*, where the delay (τ) between the excitation π/2 pulse and refocusing π pulse was one rotor period (28.5 μs). At high spinning speeds, the dipolar couplings between carbons and protons are considerably suppressed, and the proton decoupling is not necessary. Since the hyperfine coupling between the copper­(II) *d-*electron and the carbon nuclei causes fast spin–lattice relaxation, the relaxation delay between accumulations was as short as 25 ms. The temperature of the spinning sample was 315 K. The NMR shift values are given on the TMS scale. The MAS NMR spectrum was fitted using the Bruker TOPSPIN program for solid-state line shapes, where each chemically different site in the sample is given with a peak at the isotropic chemical shift accompanied by a sideband pattern, *i.e*., by a number of spinning sidebands at frequencies multiple of the sample spinning frequency from the main peak.

#### 2.6.2 DFT Calculations

The molecular geometry of compound **1** was obtained from XRD coordinates, and the positions of the hydrogen atoms were reoptimized using Turbomole 7.3 with the PBE0 functional and the def2-TZVPP basis set in vacuo. The EPR parameters (electronic g-tensor and hyperfine A-tensors) were calculated at the two-component relativistic DFT level of theory (SO–ZORA, zeroth-order regular approximation with spin–orbit coupling) with the PBE0 wave function, the TZ2P basis set, and the COSMO model of water implemented in the ADF2024 package. Hyperfine ^13^C NMR shifts ( $\delta_{L}^{\mathrm{hf}}$ ) were calculated from the EPR parameters as previously reported.^46–48^ Orbital ^13^C NMR shifts ( $\delta_{L}^{\mathrm{orb}}$ ) were calculated using the ZORA approach and were referenced to benzene, used as a secondary reference (128 ppm). The total NMR shift of a ligand atom L ( $\delta_{L}^{\mathrm{tot}}$ ) was calculated as the sum of orbital and hyperfine contributions (see Eq. (2)):

2 $$\delta_{L}^{\mathrm{tot}}=\delta_{L}^{\mathrm{orb}}+\delta_{L}^{\mathrm{hf}}$$

The spin density for **1** (see Section 3) was calculated at the scalar-relativistic DFT level of theory in the ADF2024 program.

## 3 Results and Discussion

### 3.1 Crystal Structure of **1** and Hirshfeld Surface Analysis

The asymmetric unit of **1**, {[Cu­(l–Arg)­(phen)­(*μ-*NO_3_)]­(NO_3_)·H_2_O}_n_, consists of two Cu centers coordinated by l–arg zwitterions, phen molecules, and NO_3_^–^ ions (Figure 1a). Selected bond lengths and bond angles are collected in Table S2. The pseudo chain based on O19–H191···O2 and O19–H192···O14 hydrogen interactions (D···A = 2.719(4) Å and 2.756(6) Å, respectively) links the coordination units in the asymmetric unit (Figure 1b). In the hexa-coordinated environment of Cu center, the equatorial positions are filled by carboxylate O and amino N atoms from l–Arg moieties, as well as both N nitrogen atoms from phen ligands (1.92–2.01 Å). The remaining two axial positions around the Cu centers are occupied by nitrate­(V) oxygen atoms with relatively long Cu–O distances in the range 2.42–2.76 Å. These Cu–O_NO_3__ long distances indicate the strong elongation of the octahedral geometry around the copper­(II) ions. The calculated values of the tetragonality *T* parameter (*T* = *R*_int_/*R*_out_, where *R*_int_ and *R*_out_ are the average in-plane and out-of-plane Cu–O, N distances, respectively^49^) for the [CuN_2_NOO_2_’] chromophore in the asymmetric unit are equal to 0.766 and 0.756, indicating the static character of the Jahn–Teller distortion (*T* ≤ 0.9).^50^ The nitrate ions act as a bridge linking the [Cu­(l–Arg)­(phen)]^2+^ coordination units into the one-dimensional (1D) polymer chains (Figure 1b).

**Figure 1:**
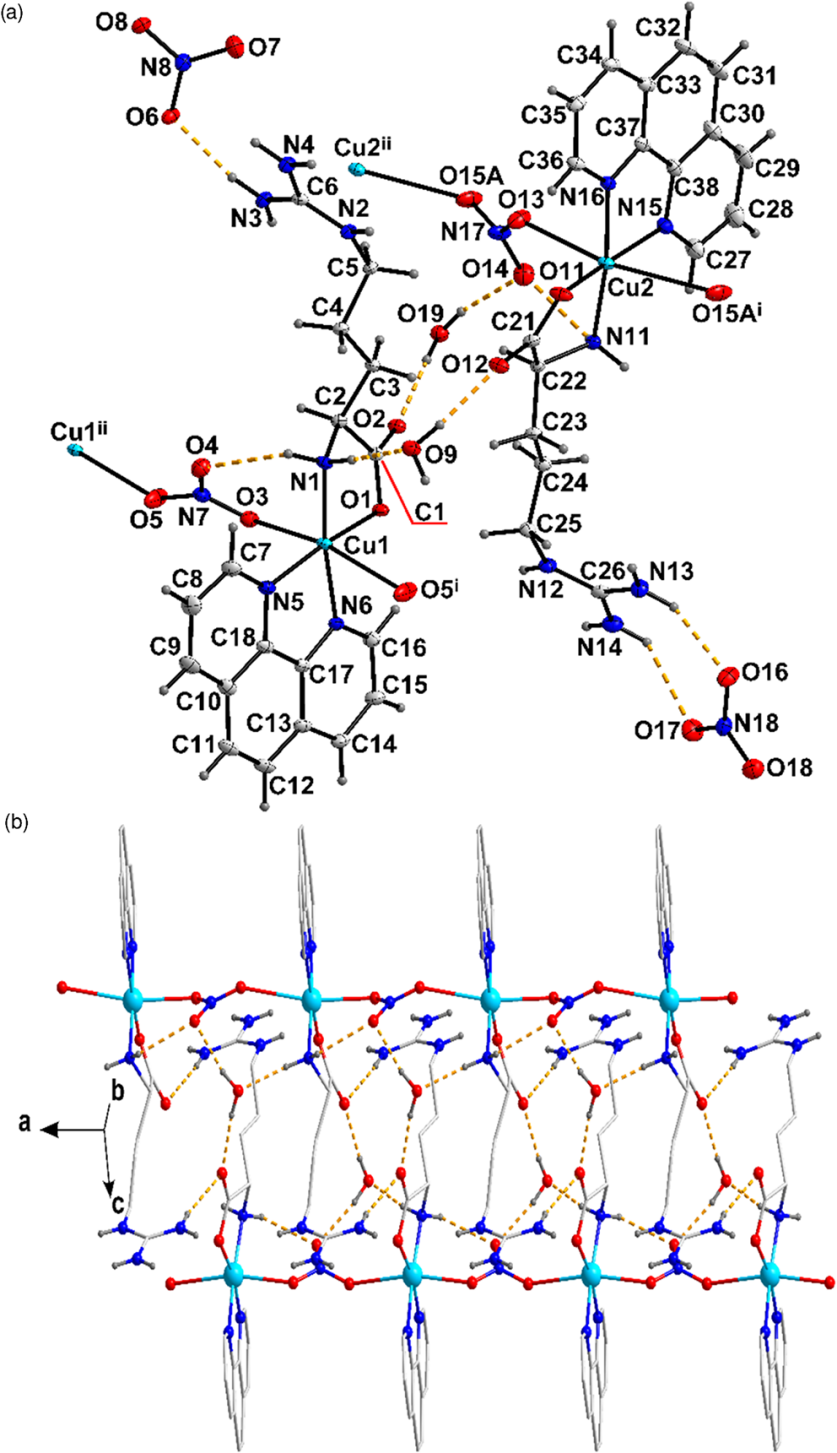
(a) Asymmetric unit; (b) 1D polymeric chain of compound **1**. Select interatomic distances (Å) Cu1–O1= 1.931(3) Å, Cu2–O11 = 1.925(3) Å, Cu1–N1 = 1.997(5) Å, Cu2–N11 = 1.989(5) Å, Cu1–N5 = 2.015(4) Å, Cu1–N6 = 2.001(5) Å, Cu2–N15 = 2.014(4) Å, Cu2–N16 = 1.994(5) Å, Cu1–O3 = 2.421(4) Å, Cu1–O5^(i)^ = 2.758(4) Å, Cu2–O13 = 2.443(4) Å, Cu2–O15A^(ii)^ = 2.755(10) Å, respectively, (i) 1+*x*,*y*,*z*; (ii) −1+*x*,*y*,*z*, the disordered O15 atom was modeled in two positions O15A and O15B, respectively.

The Cu­(II) centers within the chain are 7.071 Å apart, while the corresponding distance in the analogous compound with the bpy ligand, {[Cu­(l–Arg)­(bpy)­(*μ*–NO_3_)]­(NO_3_)}_n_, is slightly shorter (6.815 Å).^35^ In structure **1**, the next shortest Cu···Cu distances between the polymer chains are 8.448 Å, 9.005 Å, and 9.061 Å (Figure 2). The crystal structure is stabilized by a broad range of inter- and intramolecular hydrogen bonding (Figure 1). Mainly nitrogen atoms are involved as donors of N–H···O-type hydrogen bonds, similarly to the supramolecular network occurring in the structure of {[Cu­(l–Arg)­(bpy)­(*μ*–NO_3_)]­(NO_3_)}_n_.^35^ A more extensive analysis of the hydrogen-bonding system is presented in the Supporting Information.

**Figure 2:**
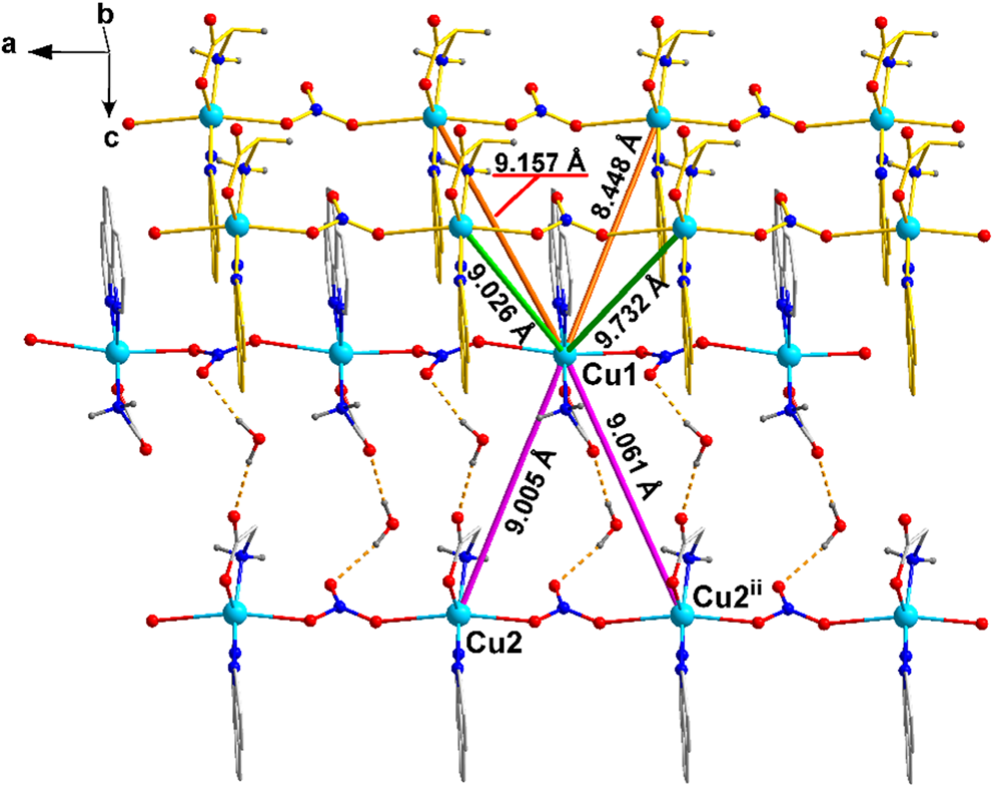
Cu···Cu distance between the polymeric chain. H atoms are omitted for clarity.

Based on Hirshfeld analysis, the two [Cu­(l–Arg)­(phen)­(NO_3_)]^+^ units in the asymmetric formula present different types of intermolecular interactions, with the molecular unit 1 showing intense red spots related to the O···H, H···H, and C···H contacts with calculated percentages of 40.2, 28.8, and 13.3%, respectively. For the molecular unit 2, the most important interactions are the O···H, N···O, N···H, and H···H contacts with the molecular packing contributions of 42.2, 1.5, 6.4, and 28.0%, respectively (Table S5). The % Cu–O interactions are calculated to be 1.5% in both units. An intense red spot labeled as “F” in the *d*_norm_ map is related to the coordination interactions between the Cu­(II) and the O5_NO_3__ and O15_NO_3__ atoms, which are responsible for the extension of the supramolecular structure into a 1D coordination polymer (Figure 3). More detailed analysis and discussion are presented in the Supporting Information.

**Figure 3:**
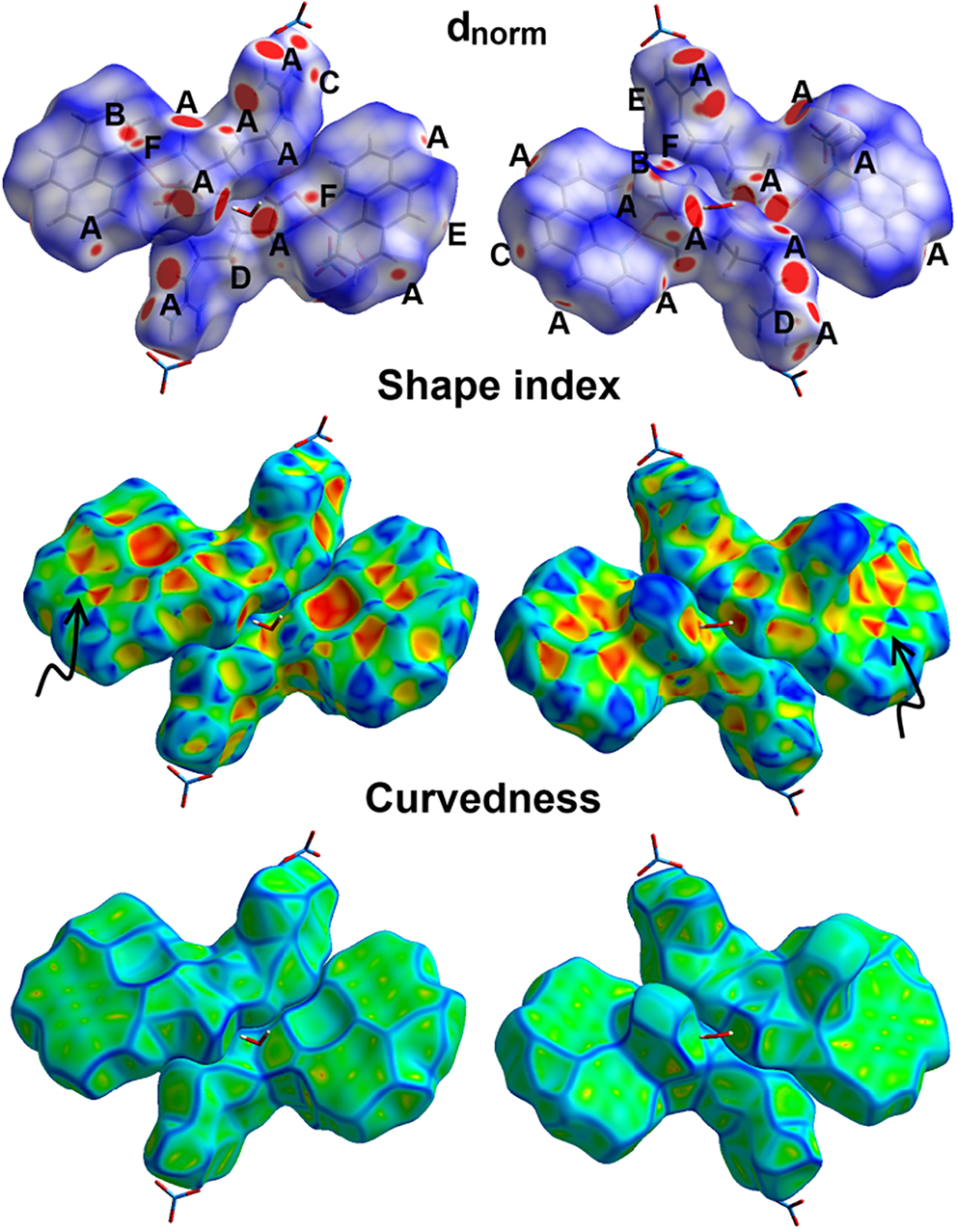
Hirshfeld surfaces of the studied complex **1**, the most important interactions are labeled as (A) O···H, (B) N···O, (C) N···H, (D) H···H, (E) C···H, and (F) Cu–O. Units 1 and 2 refer to the structures with lower and higher atom numbering, respectively.

### 3.2 Vibrational (FT-IR/FIR) and FT-Raman Spectra

The experimental infrared (IR/FIR) and Raman (R) spectra in the ranges 4000–400 cm^–1^ and 600–60 cm^–1^ of compound **1** and the ligands, *i.e*., l–Arg, phen, are presented in Figures S4–S8. In the structure of compound **1**, the NO_3_^–^ ions exist as two different entities: uncoordinated (free ion) and linked to the copper­(II) ions, forming the Cu–O–N–O–Cu bridges. The free NO_3_^–^ ion (planar, D_3h_ point group) is characterized by four fundamental modes, *i.e*., symmetric NO stretches (ν_s_(N–O), ν_1_(A_1_)), out-of-plane deformations (δ, ν_2_(A_2_)), antisymmetric stretches (ν_as_(N–O), ν_3_(E)), and in-plane deformations (δ, ν_4_(E)). Of these, modes ν_3_ and ν_4_ are IR and Raman-active, while mode ν_1_ is only Raman-active and mode ν_2_ is only IR-allowed.^51–55^ The frequency around 1370 cm^–1^ (IR), 1045 cm^–1^ (R), and 825 cm^–1^ (IR) can be assigned to the ν_3_, ν_1_ and ν_2_ vibrations of NO_3_^–^ ions having *D*_3*h*_ symmetry (Figures S4 and S5). The ν_2_ vibrations are experimentally difficult to detect in the spectrum of compound **1**, although the corresponding modes are active in the infrared. The symmetry of the planar nitrate ion is reduced to at most *C*_2*v*_ under the perturbation of the environment, *e.g*., the bidentate bridging coordination to the metal ions. The ν_1_ keeps the A symbol, but the ν_2_ becomes B, while the doubly degenerated ν_3_ and ν_4_ modes split into A + B components (Raman allowed).^53–55^ For bridged nitrates belonging to the *C*_2*v*_ point group, the band assigned to the ν_as_ fundamental ν_3_ appears as a peak at *ca*. 1295 cm^–1^ (IR) and 1308 cm^–1^ (R). The splitting of ν_3_ is used as a criterion for distinguishing different nitrate coordination modes. However, the corresponding ν_3_ modes are strong but very poorly resolved (Figure S5).

### 3.3 Magnetism

DC magnetic properties in a field of 0.5 T are presented in Figure 4, as magnetic susceptibility *vs* temperature in the range 1.8–300 K and magnetization *vs* magnetic field from 0 to 5 T. The field dependences of the magnetization from 0 to 5 T were measured at 2 K. The χ_m_ *T* product values (0.462 ± 0.005 cm^3^ K mol^–1^, μ_eff_ = 1.92 ± 0.01 μ_B_) are practically constant with decreasing temperature from 300 to 15 K. Below 15 K, a slight decrease in the value of χ_m_ *T* product, to 0.429 ± 0.005 cm^3^ K mol^–1^, is observed (μ_eff_ = 1.85 ± 0.01 μ_B_).

**Figure 4:**
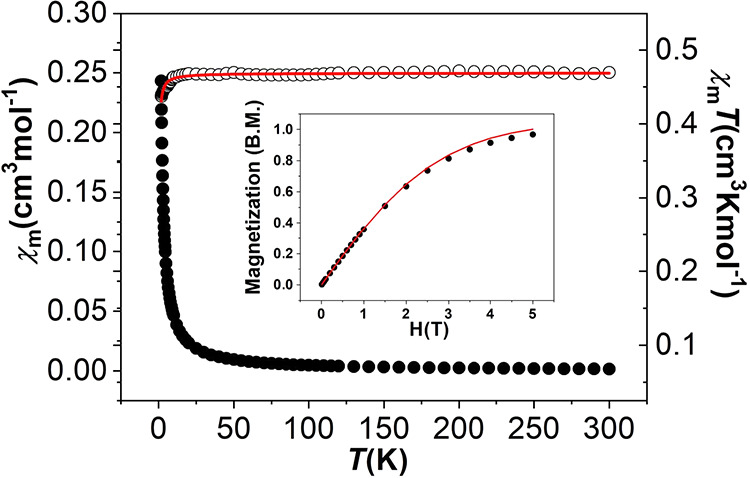
Temperature dependence of χ_m_ *T* product of **1**. The inset shows the magnetization vs applied magnetic field at 2 K. The solid lines are obtained with the parameters presented in the text, calculated using the PHI program.

The 1/χ_m_ versus *T* plots obey the Curie–Weiss law with the Curie constant *C* = 0.469 ± 0.002 cm^3^ K mol^–1^ and the Weiss constant θ = −0.23 ± 0.01 K. The small and negative value of the Weiss constant suggests weak antiferromagnetic interactions between copper­(II) ions with *S* = 1/2. The isothermal field dependence of magnetization can be well reproduced with a Brillouin function with *S* = 1/2 and *g* average values of 2.12, taken from the EPR spectra (solid line in Figure 4, inset). Taking into account crystal structure information: polymeric chain of Cu­(II) ions, through nitrate ions, connecting axial positions of copper­(II) ions, giving Cu···Cu distance equal to 7.071 Å and intermolecular bonds, through H–bonds about 8–9 Å, simulation of magnetic susceptibility curves was performed using molecular field correction model^56^: $\chi_{m}=\frac{\chi }{(\frac{zJ^{\prime }}{Ng^{2}\beta^{2}})\chi }$ , incorporated in the PHI program.^57^

The simulation yields very low parameters of magnetic interactions between nearest neighbors of *z* Cu­(II) centers: *zJ*′ = −0.22 ± 0.01 cm^–1^ and *g* = 2.20 ± 0.01, with *R* = 2.99·10^–5^. In the polymeric chain, *z* nearest neighbor is equal to 2. The magnetic properties of **1** were fitted to the model proposed by *Bonner and Fisher*,^58^ for a regular antiferromagnetic chain when *J* is negative. Received parameter *J* = −0.15 ± 0.01 cm^–1^, with *R* = 1.83·10^–5^, is comparable to those previously obtained. The fact that the observed magnetic interactions are very weak is justified by the large distances between the Cu­(II) centers. The second factor determining the character and strength of the magnetic interactions is the bridging ligand. The axial–axial bridging mode of the nitrate ion results in a symmetry-unfavorable σ–type delocalization from the copper­(II) ions. The unpaired electron on Cu­(II) ions is in the *xy* plane, leading to a small overlap and weak antiferromagnetic interactions between the Cu­(II) ions bridged by the nitrate.^56^

Compound **1** is a rare example where the nitrate ion acts as a connecting bidentate bridge in the Cu­(II) complex family. Similarly, very weak antiferromagnetic coupling has also been documented by nitrite NO_2_^–^ bridges;^59^ however, in that system, the nitrite ion connects in an axial–equatorial bridging mode.

The intermolecular interactions (H–bond and π–π stacking) existing in the crystal structure, seem to be negligible in the magnetic interactions due to the long distance between the Cu­(II) ions.

### 3.4 EPR and Electronic NIR–Vis–UV Solid-State Spectra

The spectra for complexes **1** (X-band frequencies) exhibit broad signals (Figure S9), but anisotropy of the *g* parameters is possible to be observed. The g-tensor components are typical of species with axial symmetry of the copper­(II) center (*S* = 1/2) and correspond to *g*_*z*_ = *g*_||_ ≫ *g*_*x*_ = *g*_*y*_ = *g*_⊥_ *>* 2.0023, although the correct values of the parameters are hard to establish. More information was obtained from measurements at Q-band frequencies, where not only the *g* parameter anisotropy was well resolved but also the hyperfine structure was revealed (Figure 5). However, the four hyperfine coupling lines of copper­(II) in the parallel orientation (*S* = 1/2, *I* = ^3^/_2_) were poorly resolved. Computer simulation of the experimental spectrum enabled to establish anisotropic parameters *g*: *g*_⊥_ = 2.056, *g*_||_ = 2.230, *g*_av_ = 2.120 (for DFT-calculated values 2.050, 2.056 and 2.175, see Figure S12 in SI) and anisotropic hyperfine coupling parameters *A*_⊥_ = 30·10^–4^ cm^–1^ and *A*_||_ = 194· 10^–4^ cm^–1^.

**Figure 5:**
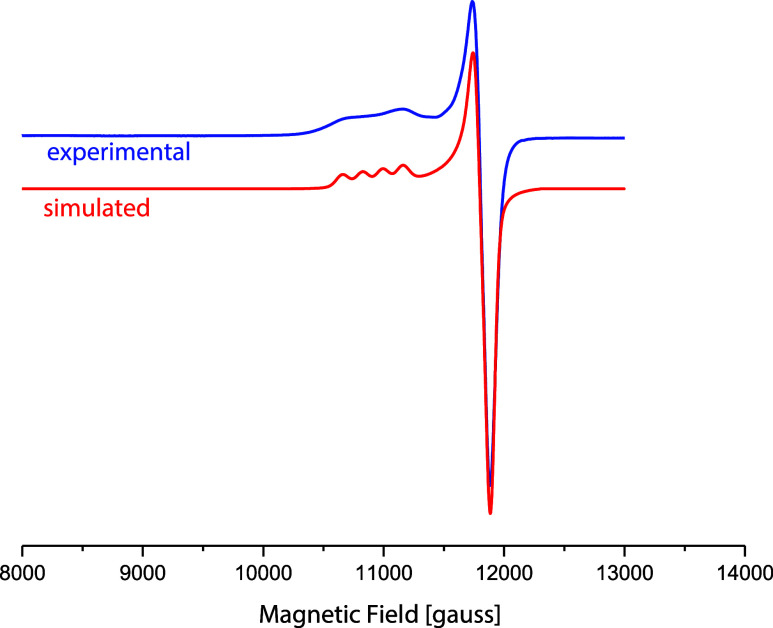
Q-band EPR spectrum of compound **1**, along with the simulated spectrum (the parameters: *g*_⊥_ = 2.056, *g*_||_ = 2.230, *A*_⊥_ = 30·10^–4^ cm^–1^, and *A*_||_ = 194·10^–4^ cm^–1^).

The parameters as well as the shape of the spectrum are very similar to those obtained for {[Cu­(*μ*–NO_3_)­(Arg)­(bpy)]­NO_3_}_n_.^35^ This indicates the d_*x*2–*y*2_ orbital of the unpaired electron (SOMO), as would be expected for the elongated O_h_ geometry of the Cu­(II) environment. The presented EPR parameters are typical for the N_3_O donor set of the ligands in the copper­(II) *xy* plane, as well as of single positive charge cationic complexes.^35,60^ The shortest Cu···Cu distance between the polymer chains of 8.44 Å (see 3.1) in the crystal structure is higher than those known for similar complexes.^35^ This phenomenon could be the reason for the revealing of the hyperfine structure due to better isolating the Cu­(II) ions and preventing exchange interactions. This EPR result stays in line with UV–vis spectra analysis.

The diffuse-reflectance spectrum of **1** presents a broad single band in the 9000–22000 cm^–1^ spectral range, which is characteristic of the *d–d*-type transition (copper­(II) ions, d^9^ configuration) (Figure S10). The spectrum is relatively similar to that found for {[Cu­(*μ*–NO_3_)­(l–Arg)­(bpy)]­NO_3_}_n_.^35^ However, the elongation of the octahedron along the Cu–O_NO_3__ bonds (*T* = 0.766/0.756) has no effect on the *d*–*d* band splitting. Only the asymmetry of the band indicates that the spectral shape consists of at least two components. Assuming the *D*_4*h*_ point group, the expected transitions are *d*_*z*2_(B_2u_) → *d*_*x*2–*y*2_, *d*_*xy*_(A_1u_) → *d*_*x*2–*y*2_ and *d_xz_* ≈ *d*_*yz*_(E_u_) → *d*_*x*2–*y*2_ (Supporting Information Scheme S1).^61^

### 3.5 ^13^C MAS NMR and DFT Calculations

NMR spectroscopy was used to characterize the in-plane distribution of the spin density in coordinated organic ligands. The ^13^C MAS NMR spectrum of **1** is shown in Figures 6 and S11, and the fitting parameters are given in Table S6. The central part of the NMR spectrum consists of the most intense peaks in an almost regular range of the ^13^C NMR shifts (Figure 6, bottom). The broad and weak peaks at sizable hyperfine NMR shifts can be seen in the 10 times magnified spectrum at the top. The weakness of these resonances in paramagnetic compounds is unsurprising. If one has a strong magnetic hyperfine field on the nucleus site, which is not well-averaged by fast magnetic exchange between paramagnetic electronic spins, one can expect the lack of signal due to the very short transverse relaxation of the nuclear spin.^62^ A similar situation was found in the case of ^13^C MAS NMR in paramagnetic acetyl acetonates,^63^ where the peaks with the highest hyperfine NMR shifts were extremely weak.

**Figure 6:**
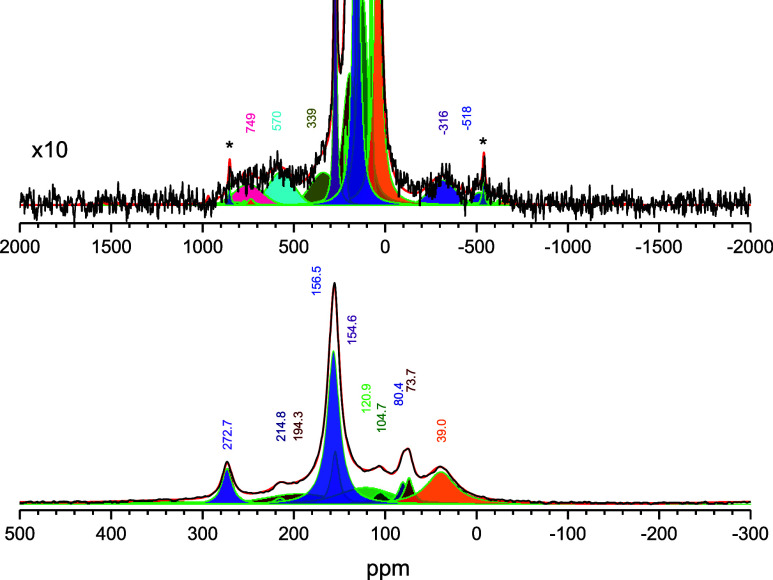
^13^C MAS NMR spectrum of compound **1** obtained with 8.7 M averages and fitted with 15 peaks given in different colors. The fitting parameters are given in Table S6. The central part of the spectrum (at the bottom) consists of the most intensive peaks in a nearly regular range of the ^13^C NMR shifts. The broad and weak peaks at sizable hyperfine NMR shifts can be seen in the 10 times magnified spectrum at the top. Due to the high spinning speed and low magnetic field, the spinning sidebands are almost missing. The two most intense sidebands belonging to the highest peak are denoted with the asterisks.

To interpret the obtained experimental ^13^C NMR shifts for compound **1** (Figure 6), we performed density functional theory (DFT) calculations (for computational details, see Section 2.6). First, we calculated the electronic g-tensor for compound **1** with in-plane components 2.05 and 2.06 and the out-of-plane component 2.17 (Figure S12). The values fit nicely with those measured experimentally (2.056 and 2.230, see Section 3.4). Although magnetic measurements revealed weak exchange coupling (see above), this interaction was neglected in all ^13^C NMR calculations. The DFT-calculated ^13^C NMR spectrum is shown in Figure 7b.

**Figure 7:**
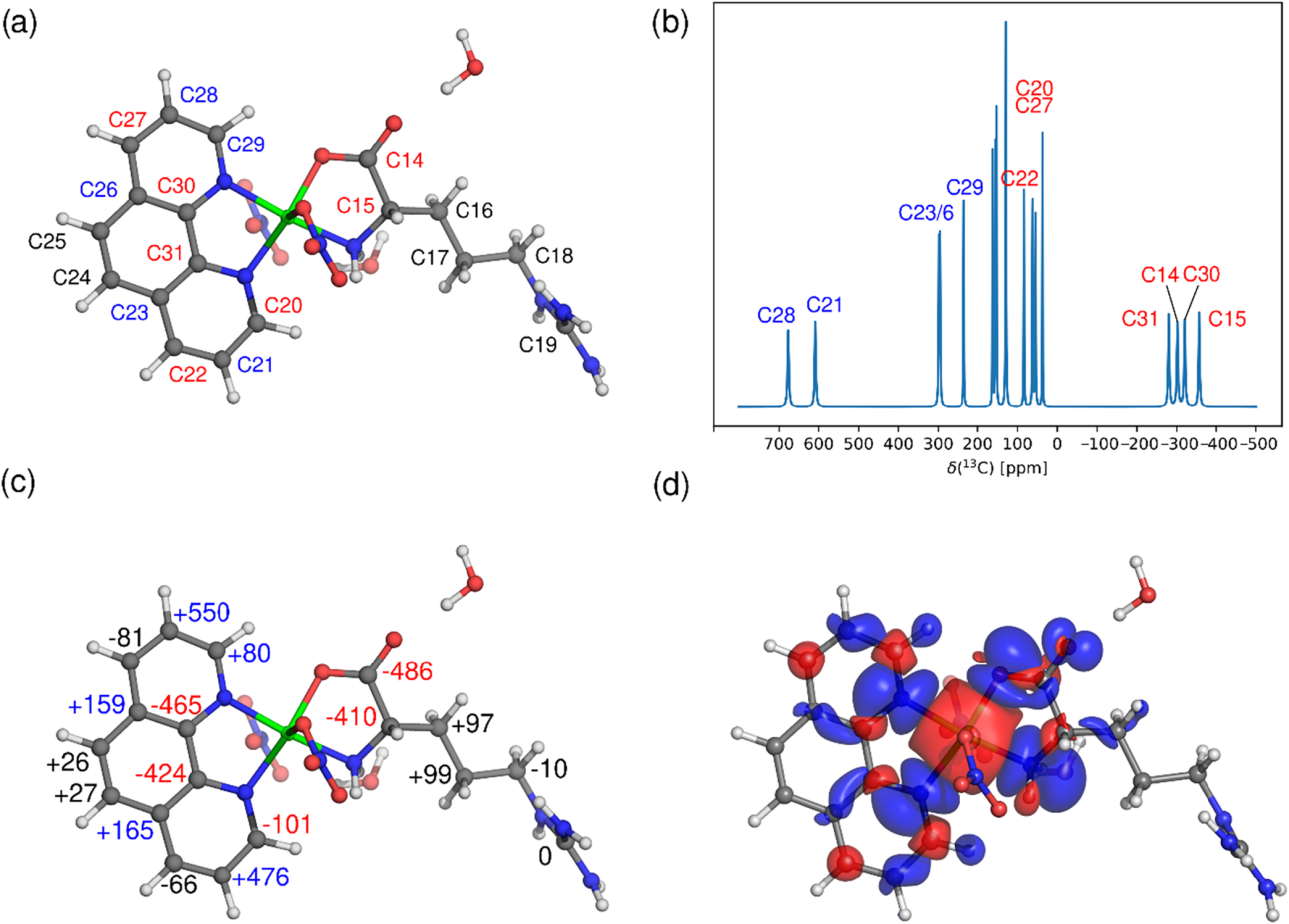
(a) Numbering scheme of carbon atoms, (b) DFT-calculated ^13^C NMR spectrum (δ^tot^ = δ^orb^ + δ^hf^) at 315 K, (c) hyperfine ^13^C NMR shifts (δ^hf^) at 315 K, and (d) distribution of spin density (isovalue 0.0002 a.u., alpha in blue, beta in red)—the source of the dominant FC contribution to δ^hf^.

Clearly, the calculated NMR spectrum is in good overall agreement with the experimental NMR spectrum. It shows that most of the signals should appear in the chemical shift range from 30 to 300 ppm, the carbons with the largest hyperfine fields should resonate at +600 to +700 ppm and −250 to −350 ppm. To interpret the extremely shielded and deshielded resonances around −300 ppm and +650 ppm, respectively, we show the hyperfine NMR shifts in Figure 7c and visualize spin density for compound **1** in Figure 7d calculated at the scalar-relativistic DFT level.^48^ The SOMO of the metal *d*_*x*2‑*y*2_-type is delocalized to three N and one O coordinating atoms (blue lobes in Figure 7d).^47,64,65^ In an aromatic ligand, this is further delocalized to the meta-carbons C21, C23, C26, and C28 (large deshielding indicated by the numbers in blue in Figure 7c). As a result of spin-polarization mechanisms from neighboring atoms,^47,64–66^ the carbon atoms neighboring the four metal-coordinating heteroatoms are negatively polarized, as shown by red lobes (significant shielding shown by red numbers in Figure 7c). Out of these eight highly paramagnetically influenced resonances, only two of C23 and C26 at +273 ppm (calculated +299 and +296) have been observed in the experimental ^13^C NMR spectrum (Figure 6, bottom). Therefore, we performed an additional ^13^C NMR experiment.

In the shielded region of this new spectrum (Figure 6, top), a very broad NMR signal at approximately −316 ppm has been identified, which corresponds to the carbons C14, C15, C30, and C31 with calculated total NMR shift values between −280 and −360 ppm (Figure 7b, Table S7). The remaining two highly deshielded carbons were predicted to resonate at +609 ppm (C21) and +678 ppm (C28). Indeed, two resonances were found in the newly performed experiment with large NMR shifts of +570 ppm and +749 ppm (Figure 6, top). In summary, we achieved a very good correlation between the calculated and experimental ^13^C NMR shifts, which allowed a detailed interpretation of the distribution of spin density in molecule **1** shown in Figure 7d.

## 4 Conclusions

The aim of our experimental and theoretical studies was the determination of the molecular structure and the intermolecular arrangement in {[Cu­(l–Arg)­(phen)­(*μ*–NO_3_)]­(NO_3_)·H_2_O}_n_ (**1**) and correlating them with the spectroscopic and magnetic properties. In this structure, the [Cu­(l–Arg)­(phen)]^2+^ cation complexes are connected via the nitrate­(V) ions to form a 1D polymer chains with a Cu···Cu distance of 7.071 Å. The bridging function of NO_3_^–^ ions was also confirmed by a strong band at *ca*. 1295 cm^–1^ (IR)/1308 cm^–1^ (R) generated by the ν_as_(N–O) (ν_3_(E)) vibrations. In the asymmetric unit, the [Cu­(l–Arg)­(phen)­(*μ–*NO_3_)]^+^ coordination entities are joined by a pseudo chain based on O19–H191···O2 and O19–H192···O14 hydrogen interactions. The donor N_2_NOO_2_’ atoms form the static distorted JT elongated octahedral geometry around copper­(II) centers (*T* = 0.766/0.756).

The EPR and UV–vis parameters indicate the elongated octahedral geometry of the Cu­(II) coordination sphere with a *d*_*x*2‑*y*2_ orbital of unpaired electron ground state. This is typical for the N_3_O chromophore in the copper­(II) *xy* plane. The broad and asymmetric single *d–d*-type band (16970 cm^–1^) consists of *d*_*xz*_ ≈ *d*_*yz*_(E_u_); *d*_z2_(B_2u_); *d*_*xy*_(A_1u_) → *d*_*x*2–*y*2_ transitions. The long Cu···Cu distance between copper centers of 7.071 Å, through NO_3_^–^ axial–axial bridges in the polymeric chain, is the reason for only weak antiferromagnetic coupling. The DFT-calculated principal components of the electronic g-tensor reproduce nicely the experimentally obtained values. The calculated hyperfine coupling A-tensors were used to construct hyperfine NMR shifts for ligand carbon atoms. The total calculated ^13^C NMR shifts were used to partially assign the experimental ^13^C MAS NMR spectrum and analyze the NMR data. This allowed us to further interpret the distribution of spin density in the plane of the aromatic ligand.

The investigated coordination polymer belongs to a class of materials with potential for further development and applications in such areas of science and technology as spintronics. This requires further optimization of the molecular structure and supramolecular bridges to obtain a stable antiferromagnetically coupled phase.

## Supplementary Material



## Notes

Comparison of the experimental XPRD pattern with the simulated pattern, crystal data, bond lengths and angles, hydrogen-bonding parameters, Hirshfeld surface fingerprint plots and intermolecular contacts, experimental ATR FT–IR, FT–FIR, and Raman spectra, EPR X-band and NIR–Vis–UV diffuse-reflectance electronic spectra, comparison of the measured ^13^C MAS NMR spectrum (exp) to the calculated one (calc), principal components of the electronic g-tensor calculated using density-functional theory (SO-ZORA/PBE0/TZ2P), parameters of the fitted peaks in the NMR spectrum: δ­(iso), Gaussian broadening in Hz; and DFT-calculated ^13^C NMR shifts of carbon atoms (PDF)
