# Supplementary material for: Solid-State Arrangement and Magnetic Properties of Hybrid Copper(II)-L-Arginine 1D Coordination Polymer Based on Nitrate Bridges
Source: Cryst Growth Des. 2026 Jul 10;26(15):5895–904. doi: 10.1021/acs.cgd.6c00298 (PMC13449762; doi:10.1021/acs.cgd.6c00298)
Supplement: Supplementary file 1 [file cg6c00298_si_001.pdf]

# Supporting Information

## Solid-state arrangement and magnetic properties of hybrid copper(II)-L–arginine 1D coordination polymer based on nitrate bridges

Agnieszka Wojciechowska†*, Waldemar Maniukiewicz‡, Lesław Sieroń‡,

Saied M. Soliman§, Maria Jerzykiewicz¶, Maria Korabik¶, Ivo Heinmaa$, Joosep Link$,

Raivo Stern$, Jan Novotný#,£, Radek Marek#,£

*†Faculty of Chemistry, Wroclaw University of Science and Technology, Wyb. Wyspiańskiego 27, 50–370 Wroclaw, Poland.*

*‡Institute of General and Ecological Chemistry,* Łódź *University of Technology, 90–924 Łódź, Żeromskiego 116, Poland.*

*§Department of Chemistry, Faculty of Science, Alexandria University, P.O. Box 426, Ibrahimia, Alexandria 8 21321, Egypt.*

*¶Faculty of Chemistry, University of Wrocław, Joliot–Curie 14, 50–383 Wrocław, Poland*

*$Department of Chemical Physics, National Institute of Chemical Physics and Biophysics, Akadeemia tee 23, EE–12618 Tallinn, Estonia.*

*#CEITEC - Central European Institute of Technology and £ National Center for Biomolecular Research, Faculty of Science, Masaryk University, CZ–625 00 Brno, Czechia.*

* Corresponding author: Agnieszka Wojciechowska,

Faculty of Chemistry,

Wrocław University of Science and Technology, Wyb. Wyspiańskiego 27, 50–370 Wrocław, Poland

Phone: +48 71 320 20 88; E-mail: agnieszka.wojciechowska@pwr.edu.pl

**Contents**

**Figure S1.** Comparison of the experimental XPRD pattern with the simulated pattern generated from the single crystal data for compound **1**.

**Figure S2.** Decomposed fingerprint plots for the short contacts in the studied complex.

**Figure S3.** Intermolecular contacts and their percentages in the studied complex.

**Figure S4.** The experimental a) ATR FT−IR and b) Raman spectra of compound **1**

**Figure S5.** Experimental spectra in the range 1800–400 $cm^{-1}$ a) FT–IR of compound **1** and pure ligands (L−Arg and phen) and b) Raman spectrum of compound **1**.

**Figure S6**. FT−Raman spectra of ligands a) pure L−arginine and b) phen.

**Figure S7.** The spectral range 600−50 $cm^{-1}$ of a) ATR FT−FIR spectra for L−Arg, phen and compound **1**; b) Raman spectrum for compound **1**.

**Figure S8.** The spectral range 600−50 $cm^{-1}$ of Raman spectra recorded for a) L−arg, b) phen.

**Figure S9.** The comparison of the EPR X−band spectra of polycrystalline sample of compound **1** a) at room temperature (RT) in the range 0–5000 Gauss b) at room temperature (RT) in the range 2500–3700 Gauss and c) at 77K (LN) in the range 2600–3700 Gauss.

**Figure S10.** NIR−Vis−UV diffuse−reflectance electronic spectra of solid−state complex **1**

**Figure S11.** Comparison of the measured $^{13}C$ MAS NMR spectrum (exp) to the calculated one (calc). The asterisks denote the spinning sidebands.

**Figure S12.** The principal components of the electronic **g**-tensor calculated using density-functional theory (SO-ZORA/PBE0/TZ2P).

**Table S1**. Crystal data and structure refinement parameters for compound **1**.

**Table S2**. Selected bond lengths (Å) and bond angles (°) for compound **1**.

**Table S3.** Hydrogen bonding parameters (Å, °) for compound **1**.

**Table S4.** Parameters for C−H···O and C−H···N (Å, °) hydrogen bonding.

**Table S5.** Short contacts (in Å) and their corresponding interaction distances.

**Table S6.** The parameters of the fitted peaks in the spectrum: $\delta$(iso), the isotropic chemical shift in ppm; $\delta$(CSA), the chemical shift anisotropy in ppm; $\eta$(CSA), the asymmetry of the chemical shift tensor; LB, the Lorenzian broadening in Hz; GB, the Gaussian broadening in Hz; the relative integral of the peak.

**Table S7**. The DFT-calculated $^{13}$C NMR shifts of carbon atoms in **1** at 315 K.

**Scheme 1.** Schematic representation of splitting of the 3d orbitals in $D_{4h}$ symmetry.

*Crystal structure of **1***

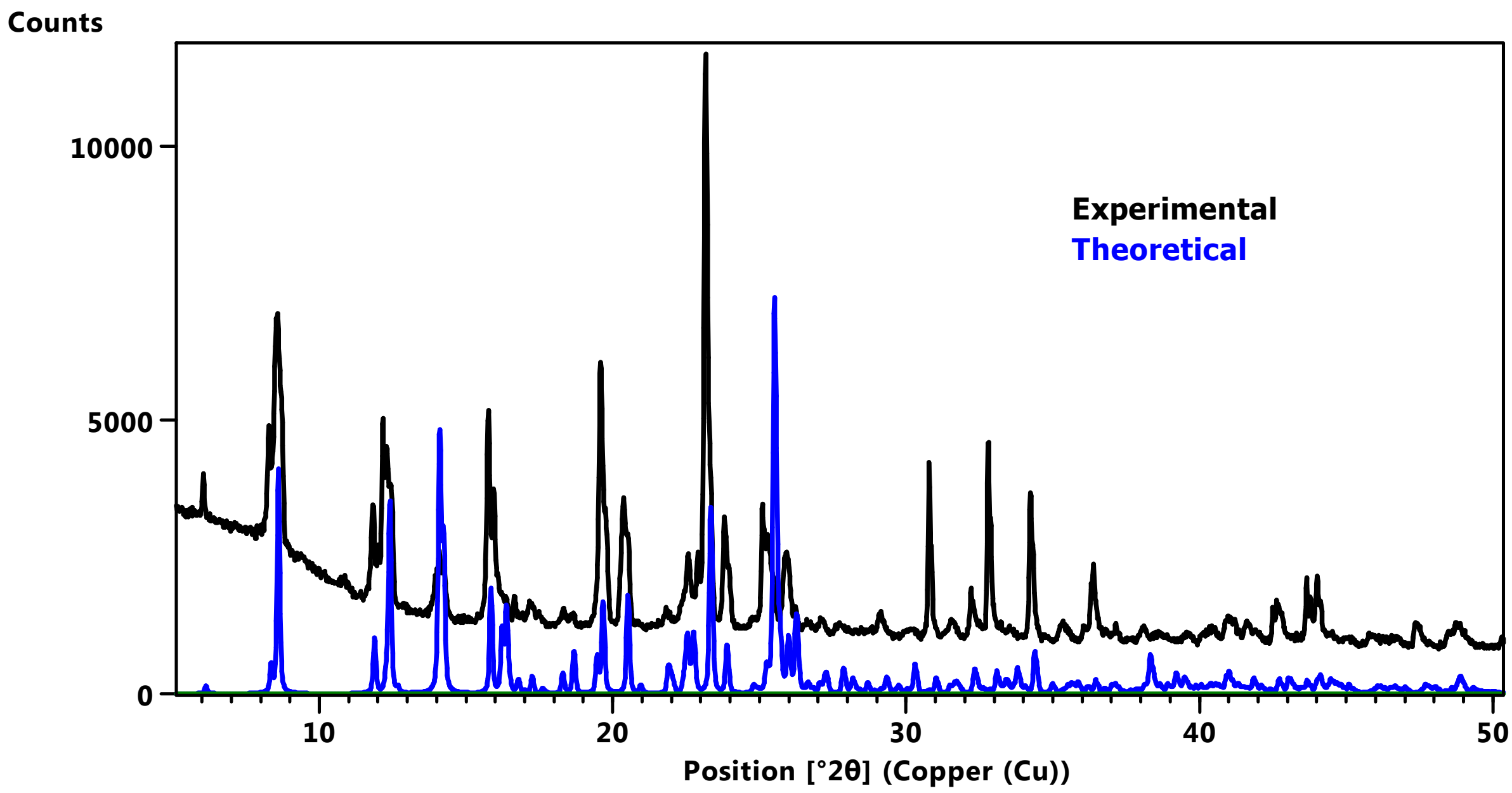


**Figure S1.** Comparison of the experimental XPRD pattern with the simulated pattern generated from the single crystal data for compound **1**.

**Table S1**. Crystal data and structure refinement parameters for compound **1**.

| Parameters | **1** |
|---|---|
| Empirical formula | $C_{18}H_{24}CuN_8O_9$ |
| Formula weight | 560.00 |
| Temperature (K) | 100 |
| Wavelength (Å) | 1.54184 |
| Crystal system | triclinic |
| Space group | P1 (No. 1) |
| a (Å) | 7.0711(1) |
| b (Å) | 11.3392(1) |
| c (Å) | 15.2632(2) |
| α (°) | 70.686(1) |
| β (°) | 86.754(1) |
| γ(°) | 80.771(1) |
| Volume ($Å^3$) | 1139.98(3) |
| Z | 2 |
| Density ($mg/m^3$) | 1.632 |
| Absorp. coeff. ( $mm^{-1}$) | 1.969 |
| F(000) | 578 |
| Crystal size ($mm^3$) | 0.04 x 0.09 x 0.29 |
| Theta range for data collection | 4.3, 70.1 |
| Index ranges | -8 <=*h*<=7 ; -13 <=*k*<13 ; -18 <=*l*<=18 |
| Reflection collected | 14020 |
| Independent reflections | 14020 |
| Goodness of fit on $F^2$ | 1.11 |
| Final R indices[I>2sigma(I)] | 0.0412 |
| Largest diff. peak and hole( $eÅ^{-3}$) | 0.39, -0.48 |

The two axial positions around the Cu centers are occupied by nitrate(V) oxygen atoms with relatively long Cu–O distances in the range from 2.421(4) Å to 2.758(4) Å (Cu1–O3= 2.421(4) Å; Cu1–O5$^{(i)}$= 2.758(4) Å and Cu2–O13= 2.443(4) Å; Cu2–O15A$^{(ii)}$ =2.755(10) Å), respectively [(i) 1+x,y,z; (ii) -1+x,y,z, the disordered O15 atom was modelled in two positions O15A and O15B, respectively].

**Table S2**. Selected bond lengths (Å) and bond angles (°) for compound **1**.

| Atoms | Bond Length (Å) | Atoms | Bond Length (Å) | Atoms | Bond Length (Å) |
|---|---|---|---|---|---|
| Cu1–O1 | 1.931(3) | Cu1–O3 | 2.421(4) | Cu2–N15 | 2.014(4) |
| Cu1–N1 | 1.997(5) | Cu1–O5$^{(i)}$ | 2.758(4) | Cu2–N16 | 1.994(5) |
| Cu1–N5 | 2.015(4) | Cu2–O11 | 1.925(3) | Cu2–O13 | 2.443(4) |
| Cu1–N6 | 2.001(5) | Cu2–N11 | 1.989(5) | Cu2–O15A$^{(ii)}$ | 2.755(10) |
| | | | | Cu2–O15B$^{(ii)}$ | 2.663(14) |

| Atoms | Bond Angle (°) | Atoms | Bond Angle (°) | Atoms | Bond Angle (°) |
|---|---|---|---|---|---|
| O1–Cu1–O3 | 86.32(13) | O3–Cu1–N6 | 91.41(15) | O11–Cu2–O15A$^{(ii)}$ | 99.6(4) |
| O1–Cu1–N1 | 84.05(16) | N1–Cu1–N6 | 172.52(18) | O13–Cu2–N11 | 93.80(16) |
| O1–Cu1–N5 | 173.05(17) | O5$^{(i)}$ –Cu1–N1 | 96.51(17) | O13–Cu2–N15 | 95.45(18) |
| O1–Cu1–N6 | 90.40(16) | O11–Cu2–O13 | 92.47(17) | O13–Cu2–N16 | 80.21(16) |
| O1–Cu1–O5$^{(i)}$ | 97.70(16) | O11–Cu2–N11 | 84.53(16) | N11–Cu2–N16 | 172.37(18) |
| O3–Cu1–N1 | 93.23(15) | O11–Cu2–N15 | 169.00(18) | O15A$^{(ii)}$ –Cu2–N11 | 92.4(4) |
| O3–Cu1–N5 | 92.69(14) | O11–Cu2–N16 | 90.98(16) | N15–Cu2–N16 | 82.89(19) |
| N5–Cu1–N6 | 82.75(18) | | | | |

Symmetry codes (i) 1+x,y,z; (ii) -1+x,y,z

In **1**, the distances between the N donor and the O acceptor atoms of N–H···O bonds vary from 2.844(6) Å to 3.218(6) Å. In contrast to the structure of $\{[Cu(\text{L}-Arg)(bpy)(\mu-NO_3)](NO_3)\}_n$,[1] the 2D and 3D supramolecular structure of **1** is additionally stabilized by O–H···O hydrogen bonds formed by water oxygen atoms (O9, O18) as donors (Table S3). Additionally, farther stabilization is supported by the oxygen water atoms as acceptor interacting with the N1 and N11 amino atoms (N1–H1B···O9; N11–H11B···$O19^{(i)}$) as well as with the N4 guanidine atoms (N4–H4A···$O19^{(iii)}$). Also, the O9 oxygen atom acting as a hydrogen bond acceptor to the uncoordinated $O4^{(i)}$ and O12 carboxylate atoms forms relatively short O9–H91···O12 (D···A =2.749(4) Å) and O9–H92···$O4^{(i)}$ (D···A = 2.779(6) Å) interactions. In structure **1** we also observe the presence of C−H···O and C−H···N weak hydrogen bonds (Table S4). Although traditionally they are not considered as strong as classic hydrogen bonds like O–H···O or N–H···O these interactions can still play significant roles in stabilizing the structure **1**. In addition to the rich hydrogen bonding, the classical weak π−π stacking interactions are also found between the neighboring phen rings with centroid−centroid distance of 3.659(3) Å.[2]

(1) Wojciechowska, A.; Rojek, T.; Misiaszek, T.; Gągor, A.; Rytlewski, P. The supramolecular hybrid inorganic–organic L-argininato-based copper(II) materials – preparation, structural, spectroscopic and thermal properties. *Inorg. Chim. Acta.* **2023**, *557*, 121698. https://doi.org/10.1016/j.ica.2023.121698

(2) Martinez, Ch. R.; Iverson, B. L. Rethinking the term "pi-stacking. *Chem. Sci.* **2012**, *3*, 2191–2201. https://doi.org/10.1039/C2SC20045G

**Table S3.** Hydrogen bonding parameters (Å, °) for compound **1**.

| D–H···A | d(D–H) | d(H···A) | d(D···A) | <(D-H···A) |
|---|---|---|---|---|
| N1–H1B···O9 | 0.91 | 1.97 | 2.874(6) | 176 |
| N2–H2N···O6$^{(i)}$ | 0.88 | 2.02 | 2.886(6) | 166 |
| N3–H3A···O11$^{(ii)}$ | 0.88 | 2.55 | 3.218(6) | 133 |
| N3–H3A···O12$^{(ii)}$ | 0.88 | 2.16 | 2.978(6) | 154 |
| N3–H3B···O6 | 0.88 | 1.98 | 2.844(6) | 167 |
| N4–H4A···O19$^{(iii)}$ | 0.88 | 2.13 | 2.935(6) | 151 |
| N4–H4B···O8$^{(i)}$ | 0.88 | 2.24 | 2.986(6) | 143 |
| N11–H11B···O19$^{(i)}$ | 0.91 | 2.00 | 2.901(6) | 169 |
| N11–H11A···O8$^{(iv)}$ | 0.91 | 2.31 | 3.074(6) | 141 |
| N12–H12N···O16$^{(ii)}$ | 0.88 | 2.00 | 2.879(6) | 172 |
| N13–H13A···O2$^{(i)}$ | 0.88 | 2.07 | 2.912(6) | 160 |
| N13–H13B···O16 | 0.88 | 2.06 | 2.855(6) | 150 |
| N14–H14A···O17 | 0.88 | 2.43 | 3.130(7) | 137 |
| N14–H14B···O18$^{(i)}$ | 0.88 | 2.30 | 3.070(7) | 145 |
| O9–H91···O12 | 0.84 | 1.91 | 2.749(4) | 173 |
| O9–H92···O4$^{(i)}$ | 0.84 | 1.95 | 2.779(6) | 168 |
| O19–H191···O2 | 0.84 | 1.89 | 2.719(4) | 171 |
| O19–H192···O14 | 0.84 | 1.93 | 2.756(6) | 167 |

Symmetry codes: (i) 1+x,y,z; (ii) -1+x,y,z ; (iii) x,-1+y,z, ( iv) 1+x,1+y,z

**Table S4.** Parameters for C–H···O and C–H···N (Å, °) hydrogen bonding.

| D–H···A | d(D–H) | d(H···A) | d(D···A) | <(D–H···A) |
|---|---|---|---|---|
| C9–H9···O14[i] | 0.95 | 2.50 | 3.280(8) | 139 |
| C14–H14···O15A[ii] | 0.95 | 2.58 | 3.438(19) | 150 |
| C23–H23A···O2 [iii] | 0.99 | 2.51 | 3.386(6) | 148 |
| C25–H25B···O1 | 0.99 | 2.46 | 3.378(6) | 155 |
| C27–H27···O8 [iv] | 0.95 | 2.56 | 3.430(7) | 152 |
| C29–H29···O4 [v] | 0.95 | 2.48 | 3.268(8) | 140 |
| C31–H31···N14[vi] | 0.95 | 2.60 | 3.517(7) | 162 |
| C34–H34···O3[vii] | 0.95 | 2.42 | 3.236(7) | 144 |
| C34–H34···O5[vii] | 0.95 | 2.44 | 3.351(8) | 160 |
| C35–H35···O7 [iii] | 0.95 | 2.52 | 3.259(7) | 135 |

Symmetry codes: (i) x,y,-1+z ; (ii) x,1+y,-1+z; (iii) 1+x,y,z ;( iv) 1+x,1+y,z; (v) x,1+y,z; (vi) x,-1+y,1+z; (vii) 1+x,-1+y,1+z;

*Hirshfeld surface analysis*

A summary of all possible supramolecular interactions with distances shorter than the sum of the vdWs radii of the interacting atoms is listed in Table S5.

**Table S5.** Short contacts (in Å) and their corresponding interaction distances.

| Contact | Distance | Contact | Distance | Contact | Distance | Contact | Distance |
|---|---|---|---|---|---|---|---|
| O6···H3B | 1.853 | O14···H192 | 1.791 | O11···H3A | 2.463 | O9···H14A | 2.408 |
| O7···H35 | 2.427 | O12···H91 | 1.771 | O8···H4B | 2.136 | O12···H4D | 2.583 |
| O2···H13A | 1.950 | O2···H23A | 2.430 | O11···H5B | 2.604 | H14B···H4C | 2.137 |
| O4···H92 | 1.811 | O1···H25B | 2.373 | O8···H27 | 2.448 | C6···H11 | 2.710 |
| O4···H1A | 2.341 | O9···H1B | 1.867 | O8···H11A | 2.237 | Cu1···O5 | 2.758 |
| O18···H1A | 2.539 | O14···H11A | 2.300 | O7···H27 | 2.583 | C12···C36 | 3.398 |
| O12···H3A | 2.044 | O5···H34 | 2.318 | O16···H12N | 1.876 | N15···H15A | 2.856 |
| O6···H2N | 1.899 | O3···H34 | 2.315 | O16···H14B | 2.591 | N14···H31 | 2.477 |
| O19···H11B | 1.907 | O15A···H14 | 2.464 | O18···H14B | 2.199 | N17···H192 | 2.443 |
| O2···H191 | 1.745 | O13···H14 | 2.504 | O16···H13B | 1.950 | Cu2···O15A | 2.755 |
| O15A···H192 | 2.608 | O14···H9 | 2.402 | O17···H14A | 2.338 | | |

The O···H, H···H and C···H contacts in unit 1 and O···H, N···O, N···H and H···H contacts in unit 2 as well as the Cu–O coordination interactions in both units appear as sharp spikes in the decomposed fingerprint plot (Figure S2).

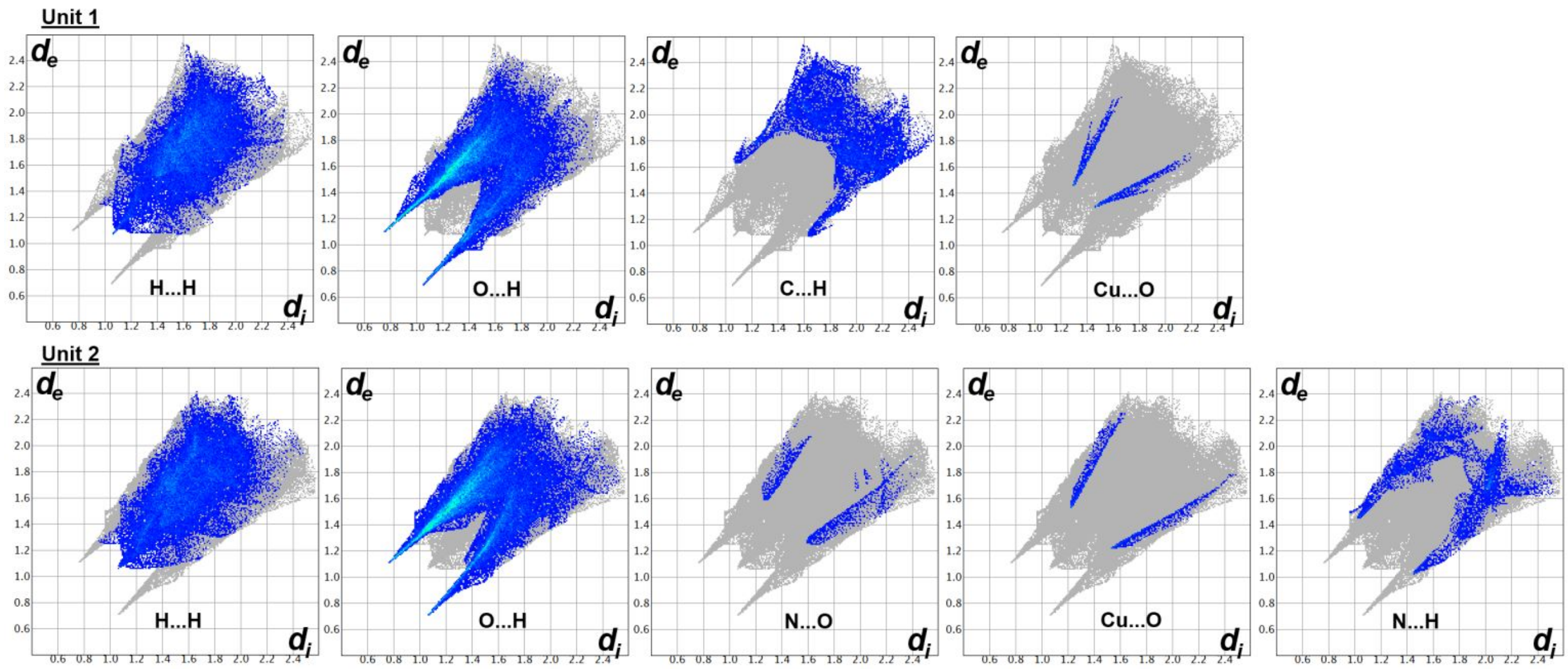


**Figure S2.** Decomposed fingerprint plots for the short contacts in the studied complex.

In addition, many other smaller and less important non–covalent contacts were detected (Figure S3). There is a short C12···C36 interaction with a contact distance of 3.398 Å, which is very close to twice the sum of the vdWs radii of the C atom (3.40 Å), indicating the presence of aromatic π–π stacking interactions. The presence of the aromatic π–π stacking interactions between the coordinated phen ligand units is further revealed by the red/blue triangles in the shape index map. For better clarity, the regions contributing to the aromatic π–π stacking interactions are indicated by the black arrow (Figure 3). The results of the Hirshfeld calculations are compared with the structurally related previously published by us $\{[Cu(\text{L–Arg})(bpy)(\mu\text{–}NO_3)](NO_3)\}_n$ complex.[1] In this case, the O···H contacts are the most important, accounting for 44.4% of the total contacts occurred in this crystal structure. In addition, the %Cu–O, which represents the interaction between the Cu(II) within the

asymmetric unit and the O atom from the $NO_3^-$ group in a neighboring complex unit, is found to contribute by 1.4% of the total contacts. No significant π–π stacking interactions are detected in this case.

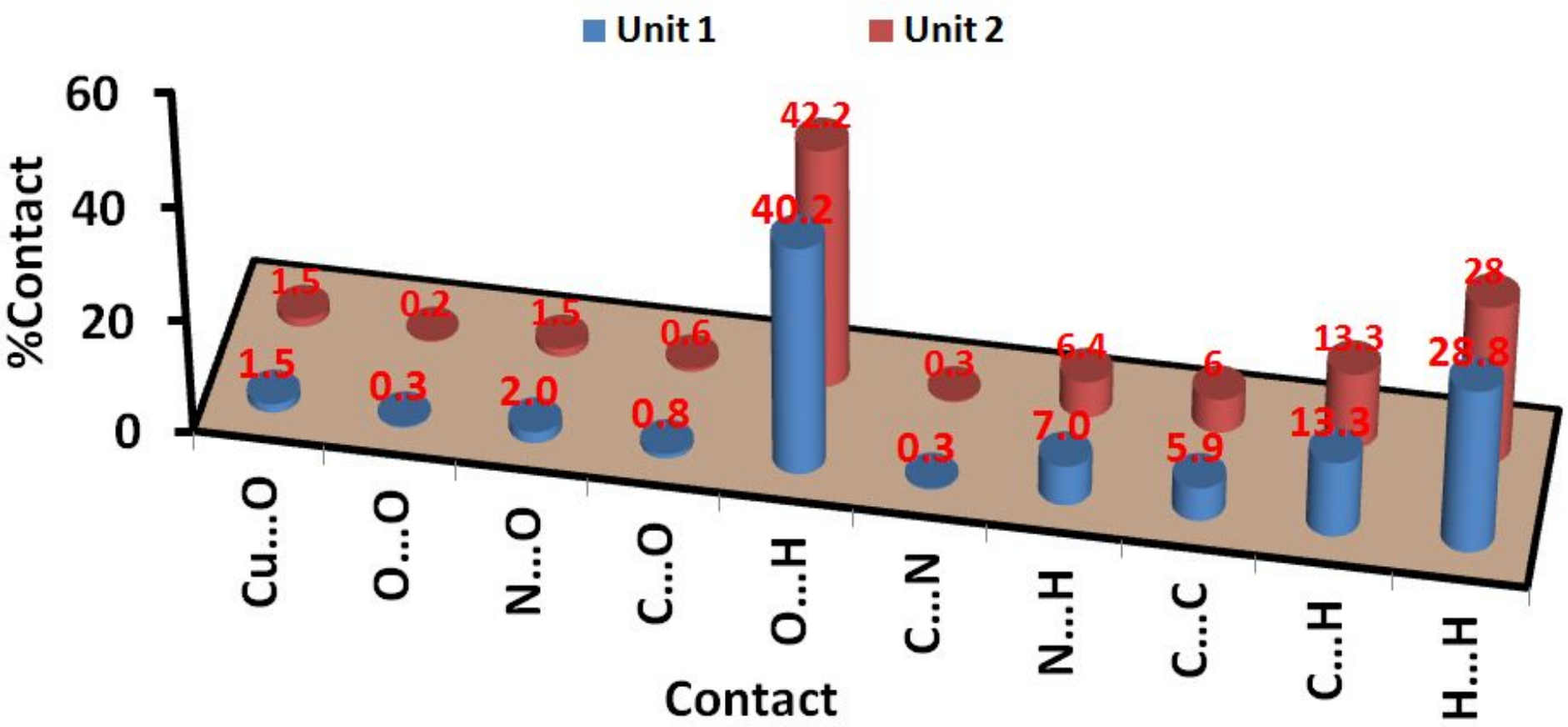


**Figure S3.** Intermolecular contacts and their percentages in the studied complex.

*Vibrational(FT-IR/FIR) and FT-Raman spectra*

The experimental infrared (IR) and Raman (R) spectra in the range 4000–400 $cm^{-1}$ of compound **1** and the ligands *i.e.* L–Arg, phen, are presented in Figures. S4- S6.

A broad absorption found in the IR spectrum (very weak in the R spectrum) for L–Arg can be attributed to ν(O–H) stretching vibrations, while for compound **1**, it is shifted to a higher frequency and appears in the range of 3500 $cm^{-1}$ to 2900 $cm^{-1}$ (Figure S4). This broad absorption of ν(O–H) overlaps the sharp and medium intensity ν(N–H), ν(C–$H_2$) and ν(C–H) vibrations. The characteristic ν($NH_2$) stretching vibrations (in the guanidinium and amino groups) appear as the weak bands at 3361 $cm^{-1}$ and 3295 $cm^{-1}$ in the Raman spectrum of L–Arg (Figure S6a). However, the corresponding vibrations of the $NH_2$ group are not found in the Raman spectrum of **1**. Relatively intense bands corresponding to the ν(C–$H_2$) and ν(C–H) stretching vibrations are observed at 2918 $cm^{-1}$ and 2862 $cm^{-1}$ for L–Arg and 2942 $cm^{-1}$, 2917 $cm^{-1}$ and 2871 $cm^{-1}$ for compound **1** in the Raman spectra (Figures S4 and S6). The very strong bands at 1455 $cm^{-1}$ (R) and 1430 $cm^{-1}$ (R) correspond to the $\delta_{sc}(CH_2)$ vibrations in compound **1**. The corresponding peaks are shifted to a lower frequency, *i.e.* 1449 $cm^{-1}$ and 1437 $cm^{-1}$ in the phen and L–arg spectra, respectively. The strong bands at 1692 $cm^{-1}$ (IR, **1**) and 1677 $cm^{-1}$ (IR, L–Arg) are attributed to the guanidinium ν(CN) vibrations. The $\nu_{as}(COO^-)$/ν(C=O) vibrations of the deprotonated carboxylate group can be assigned to a strong band at 1620 $cm^{-1}$ (IR) and 1622 $cm^{-1}$ (R). The expected $\nu_s(COO^-)$/ν(C–O) vibrations in the range of 1450 $cm^{-1}$ to 1250 $cm^{-1}$ are overlapped by the strong ν(N–O) vibrations in the $NO_3^-$ ions (Figure S4a). The very strong band at 1554 $cm^{-1}$ for L–Arg (IR) can be attributed to the scissoring $\delta_{sc}(NH_2)_{am}$ vibrations. In compound **1**, the vibrations of the coordinating $NH_2$ group have generated the band found as a shoulder at *ca.* 1640 $cm^{-1}$ (Figure S4a).

The far-infrared (FIR) and Raman spectra, below 600 $cm^{-1}$, are relatively poorly resolved (Figures S7-S8). In particular, the FIR spectrum exhibits only two main bands at 431 $cm^{-1}$ and 329 $cm^{-1}$, originating from the ν(Cu–N) and ν(Cu–O) vibrations, respectively (Figure S7). The corresponding vibrations in the Raman spectrum are found as medium intensity bands at 430 $cm^{-1}$ and 332 $cm^{-1}$. Furthermore, in the Raman spectrum of **1**, the medium intensity peaks at 558 $cm^{-1}$, 487 $cm^{-1}$, 409 $cm^{-1}$ and 295 $cm^{-1}$ can be assigned to the skeletal deformation, out-of-plane bending γ($COO^-$) modes, respectively. These modes generate Raman bands at 551 $cm^{-1}$, 488 $cm^{-1}$, 407 $cm^{-1}$ and 301 $cm^{-1}$ in the spectrum of L–Arg (Figure S7a). In the IR and Raman spectra of the phen ligand, the skeletal deformations produce bands at 410 $cm^{-1}$ (Figures S7-8b).

**a)**

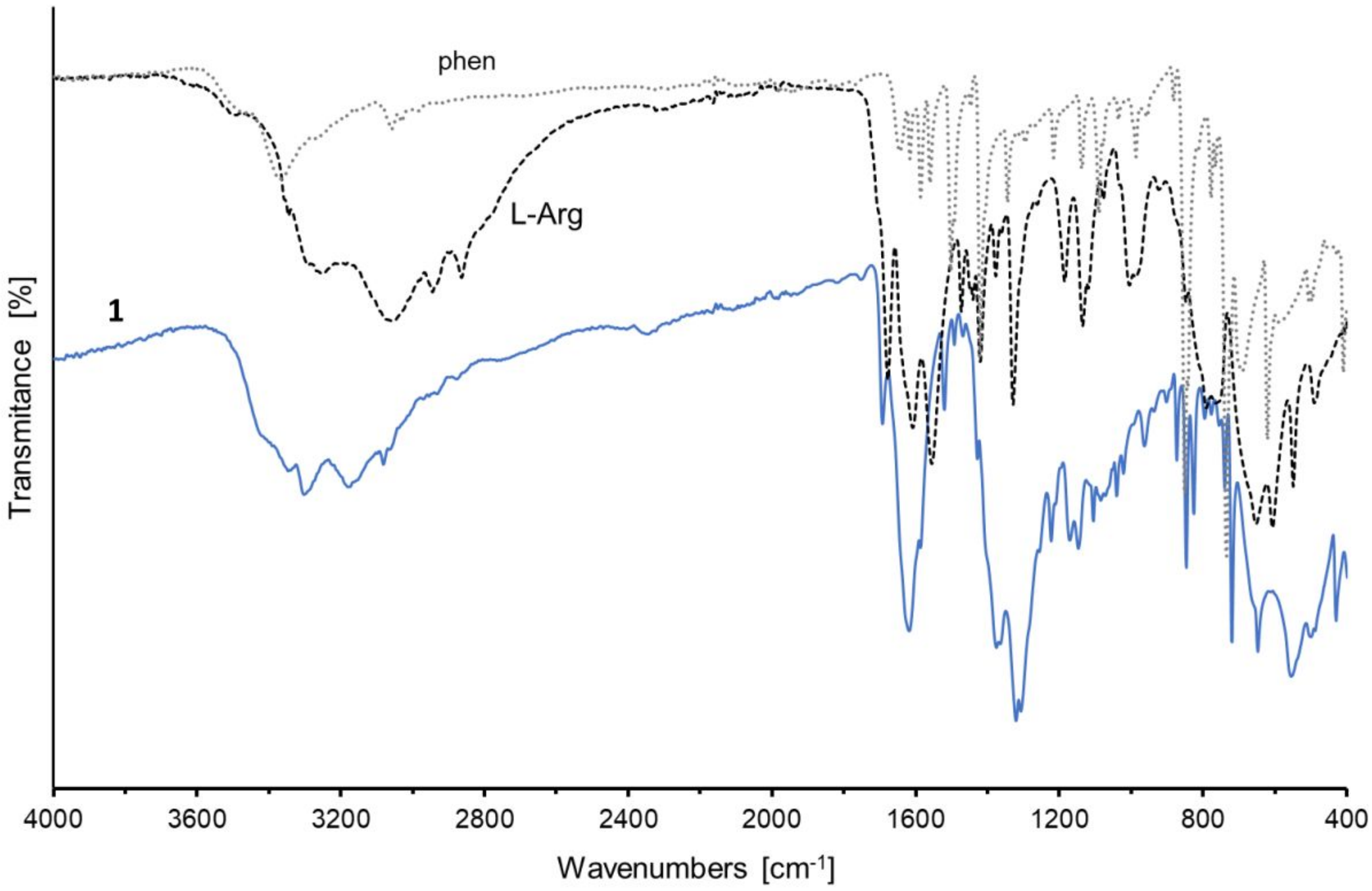


**b)**

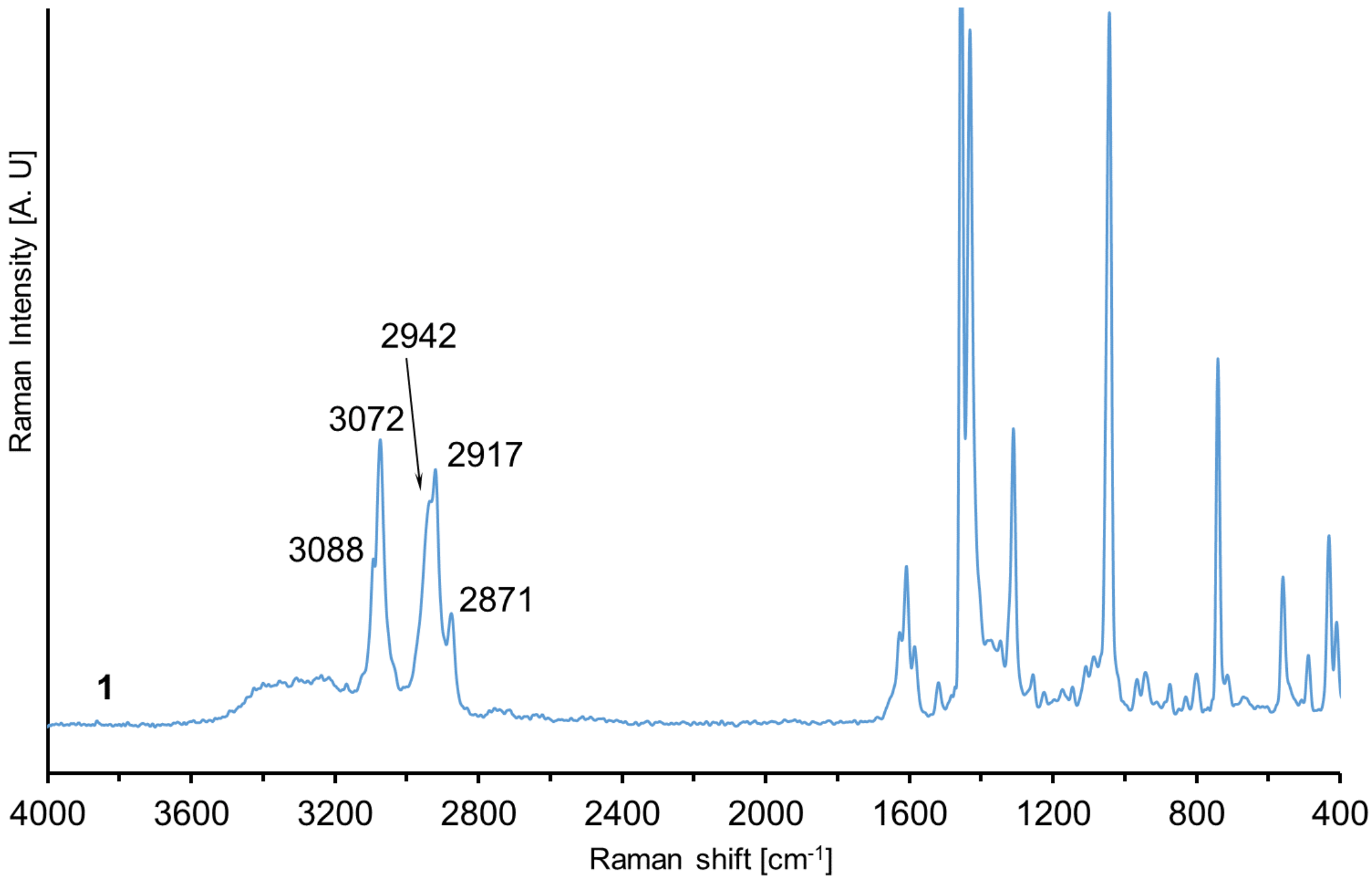


**Figure S4.** The experimental a) ATR FT–IR and b) Raman spectra of compound **1**.

a)

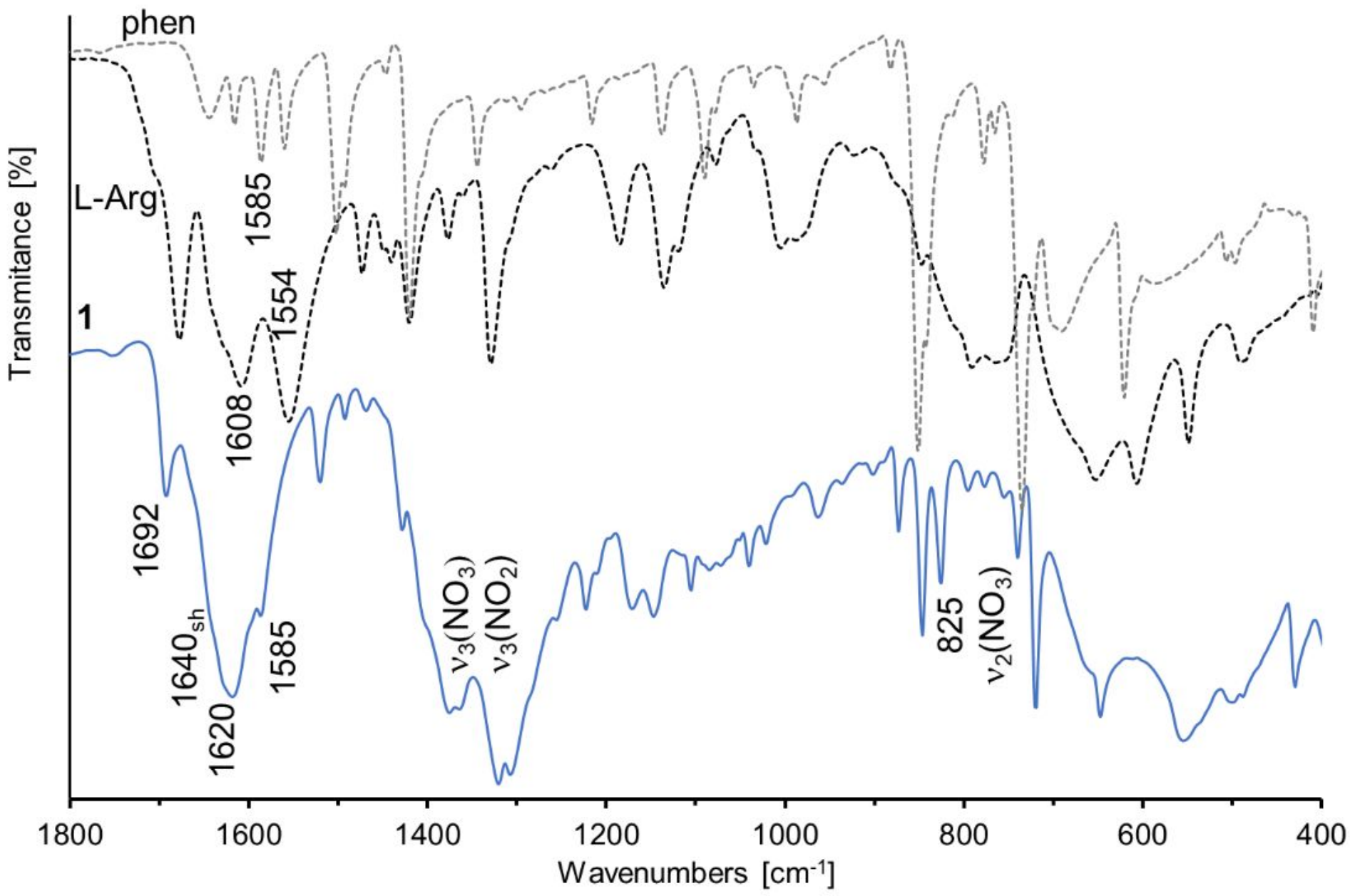


b)

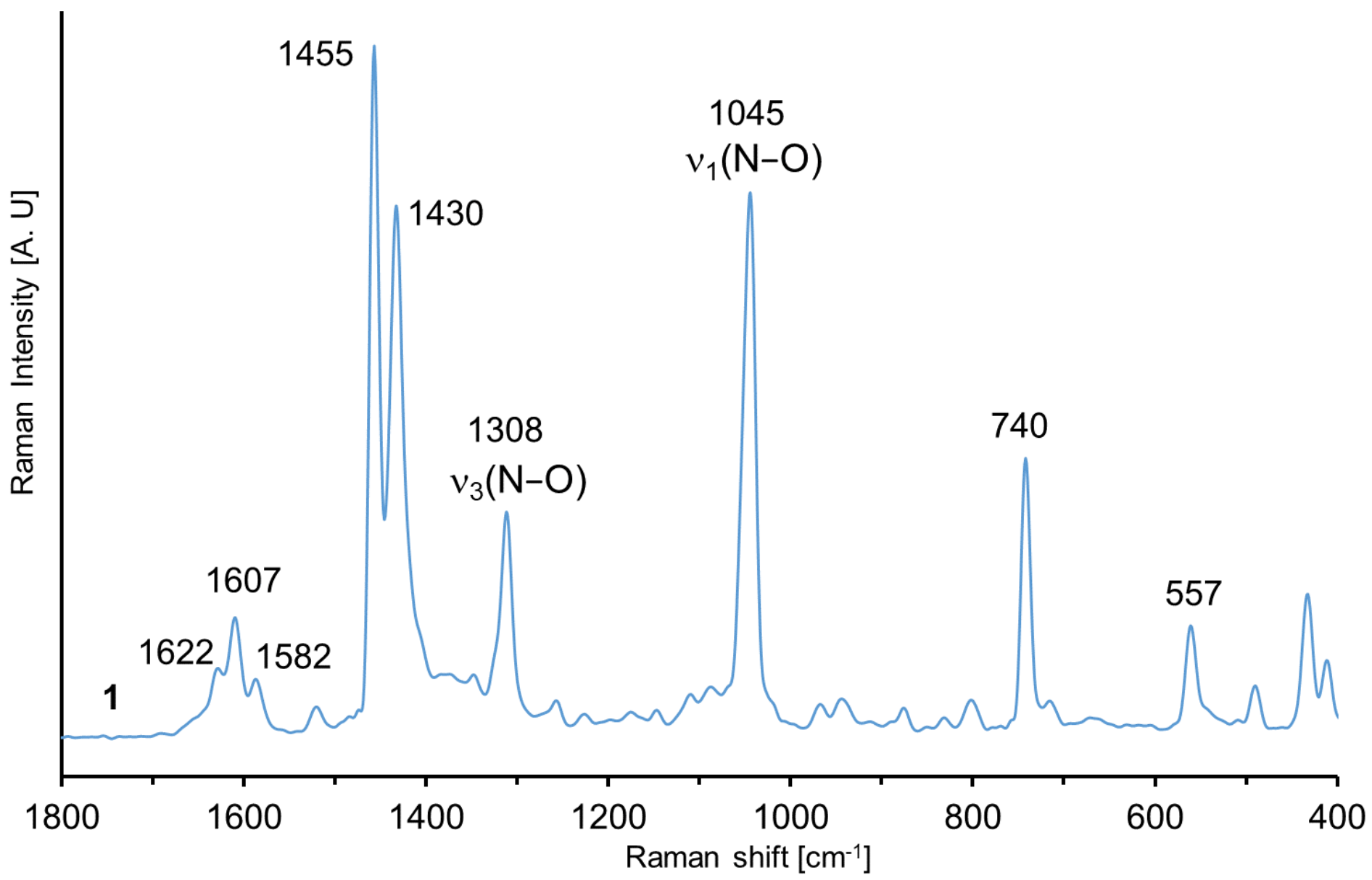


**Figure S5.** Experimental spectra in the range 1800–400 cm$^{-1}$ a) FT–IR of compound **1** and pure ligands (L–Arg and phen) and b) Raman spectrum of compound **1**.

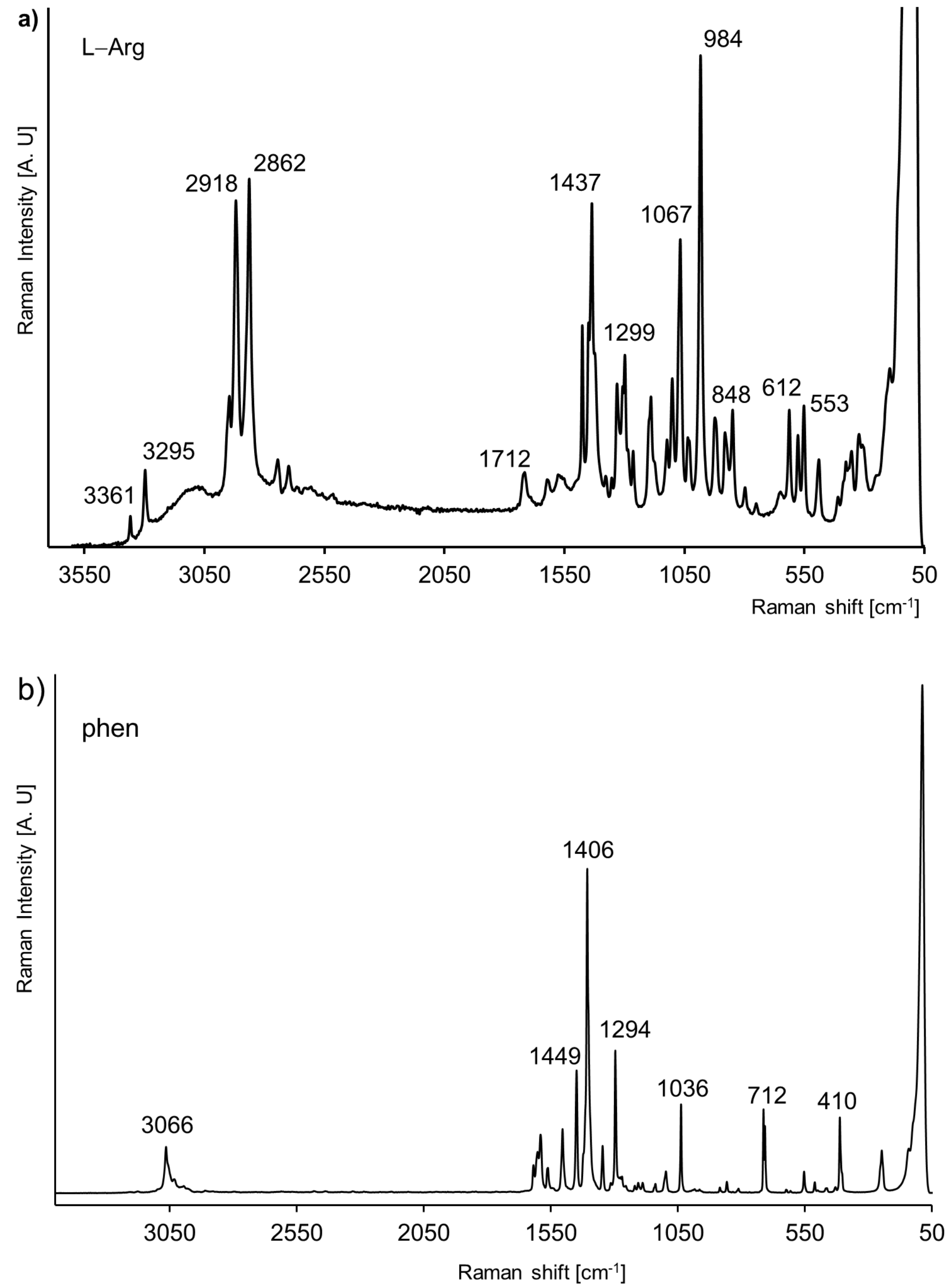


**Figure S6**. FT−Raman spectra of ligands a) pure L−arginine and b) phen.

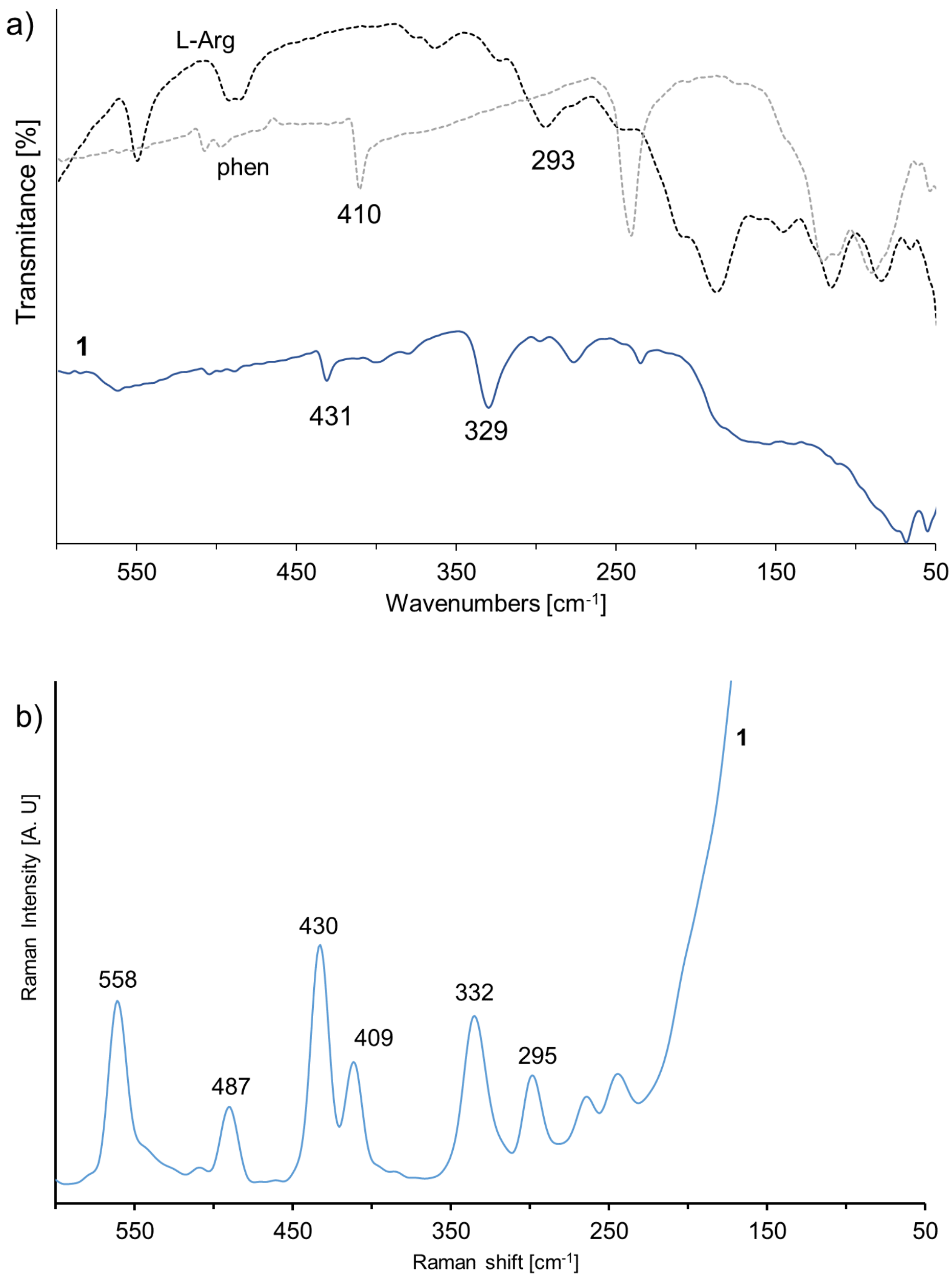


**Figure S7.** The spectral range 600−50 cm$^{-1}$ of a) ATR FT−FIR spectra for L−Arg, phen and compound **1**; b) Raman spectrum for compound **1**.

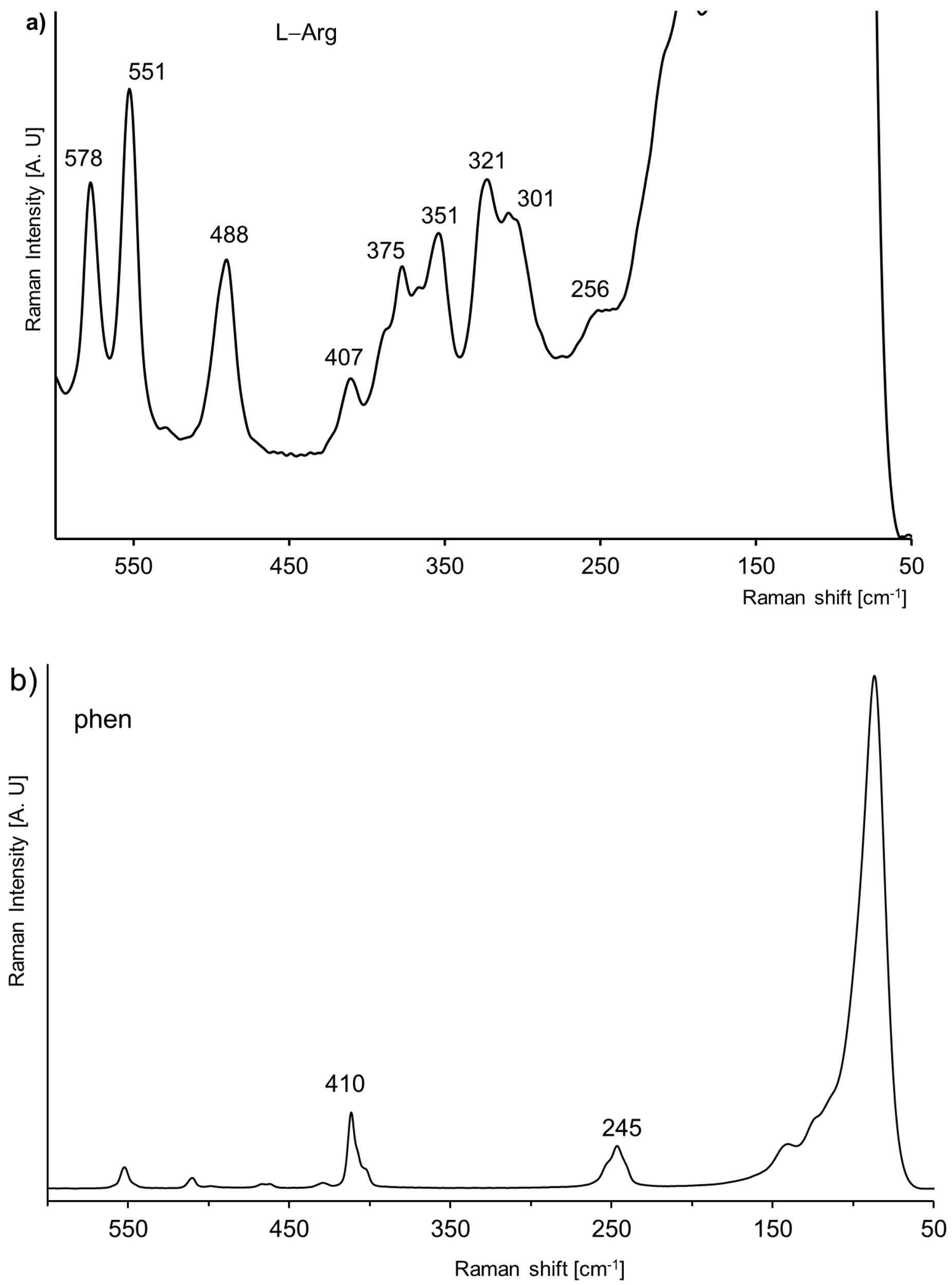


**Figure S8.** The spectral range 600−50 cm$^{-1}$ of Raman spectra recorded for a) L−arg, b) phen.

*EPR and electronic NIR-Vis-UV solid state spectra*

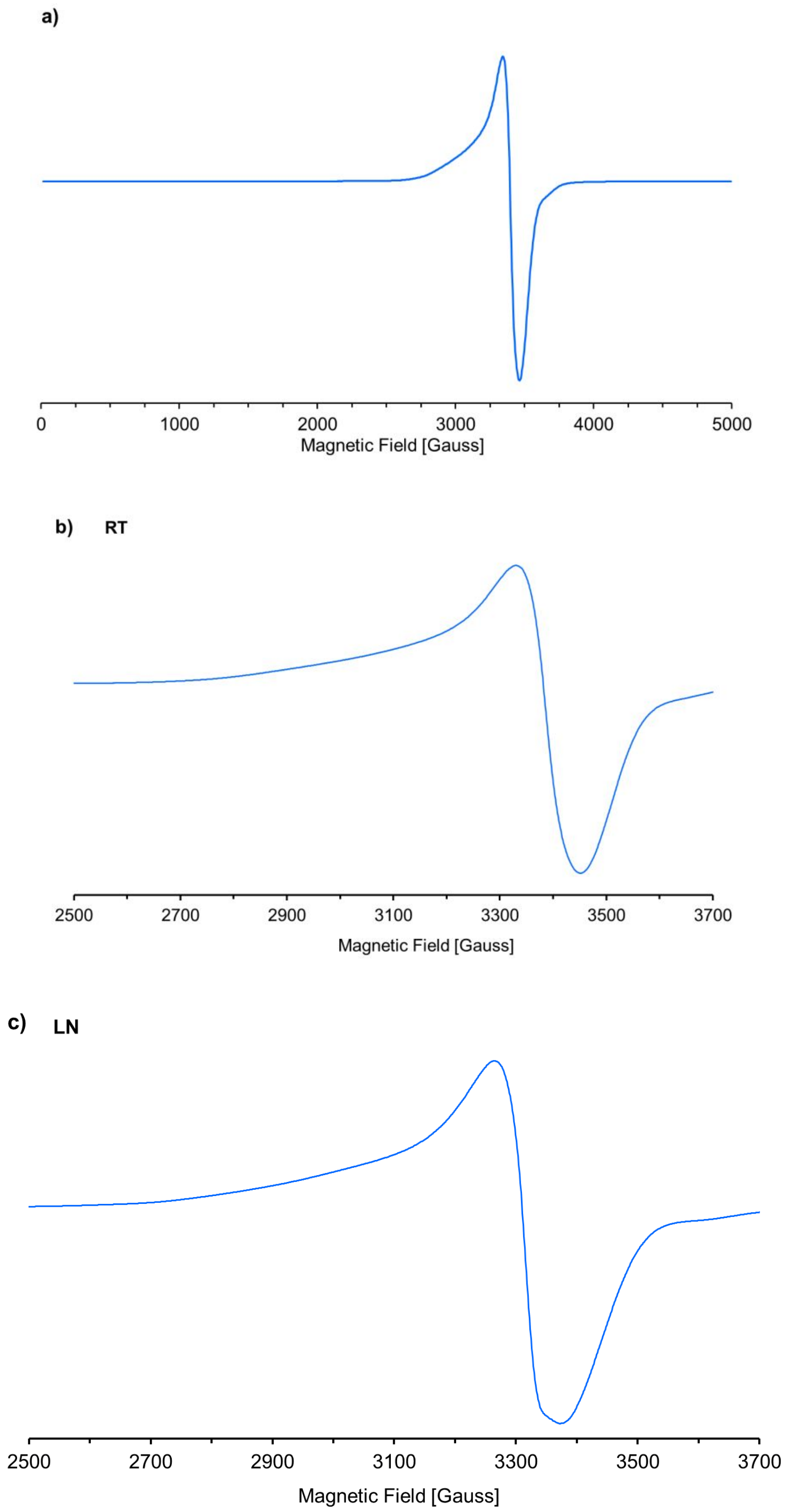


**Figure S9.** The comparison of the EPR X–band spectra of polycrystalline sample of compound **1** a) at room temperature (RT) in the range 0–5000 Gauss b) at room temperature (RT) in the range 2500–3700 Gauss and c) at 77K (LN) in the range 2600–3700 Gauss.

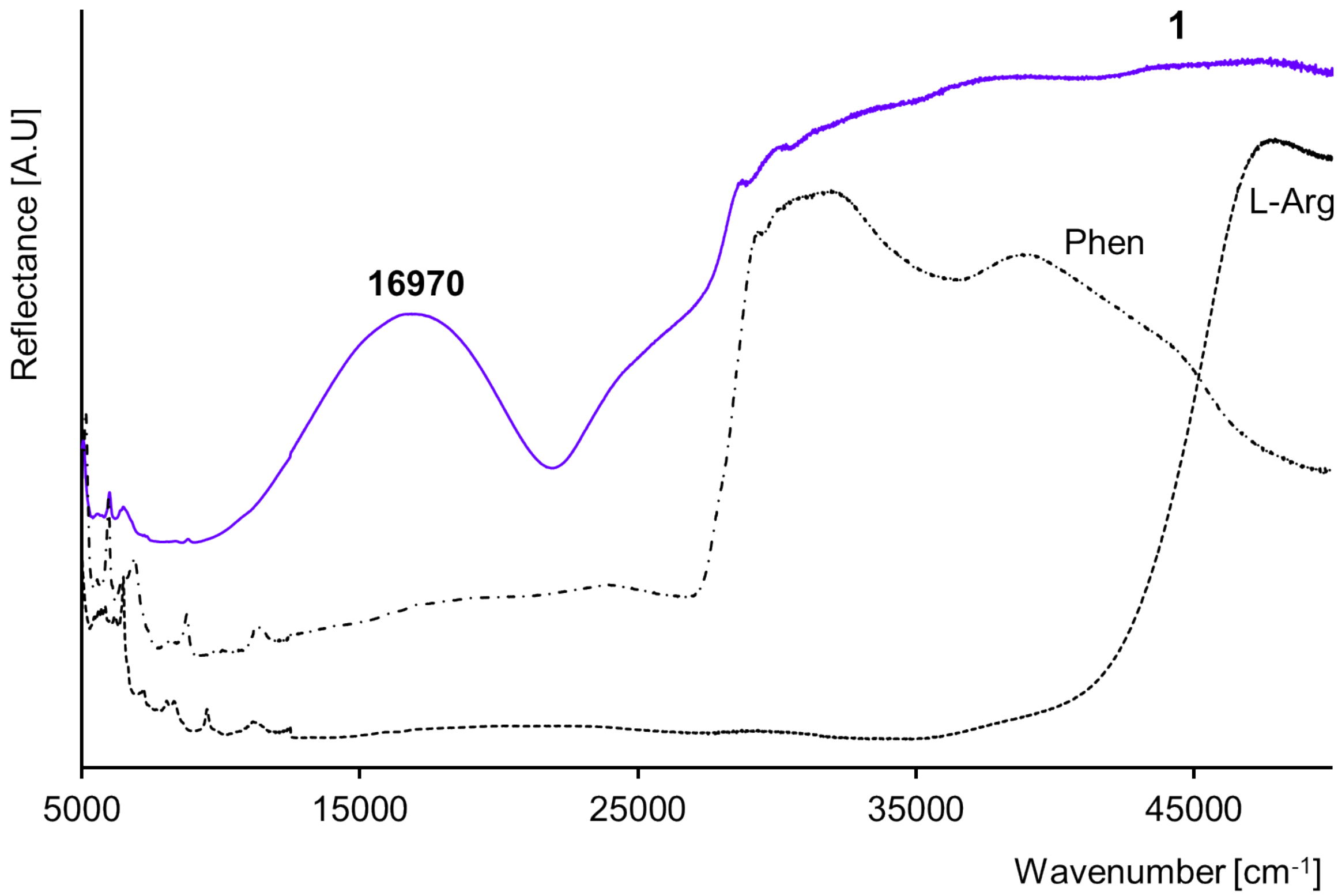


**Figure S10.** NIR–Vis–UV diffuse–reflectance electronic spectra of solid–state complex **1** and ligands.

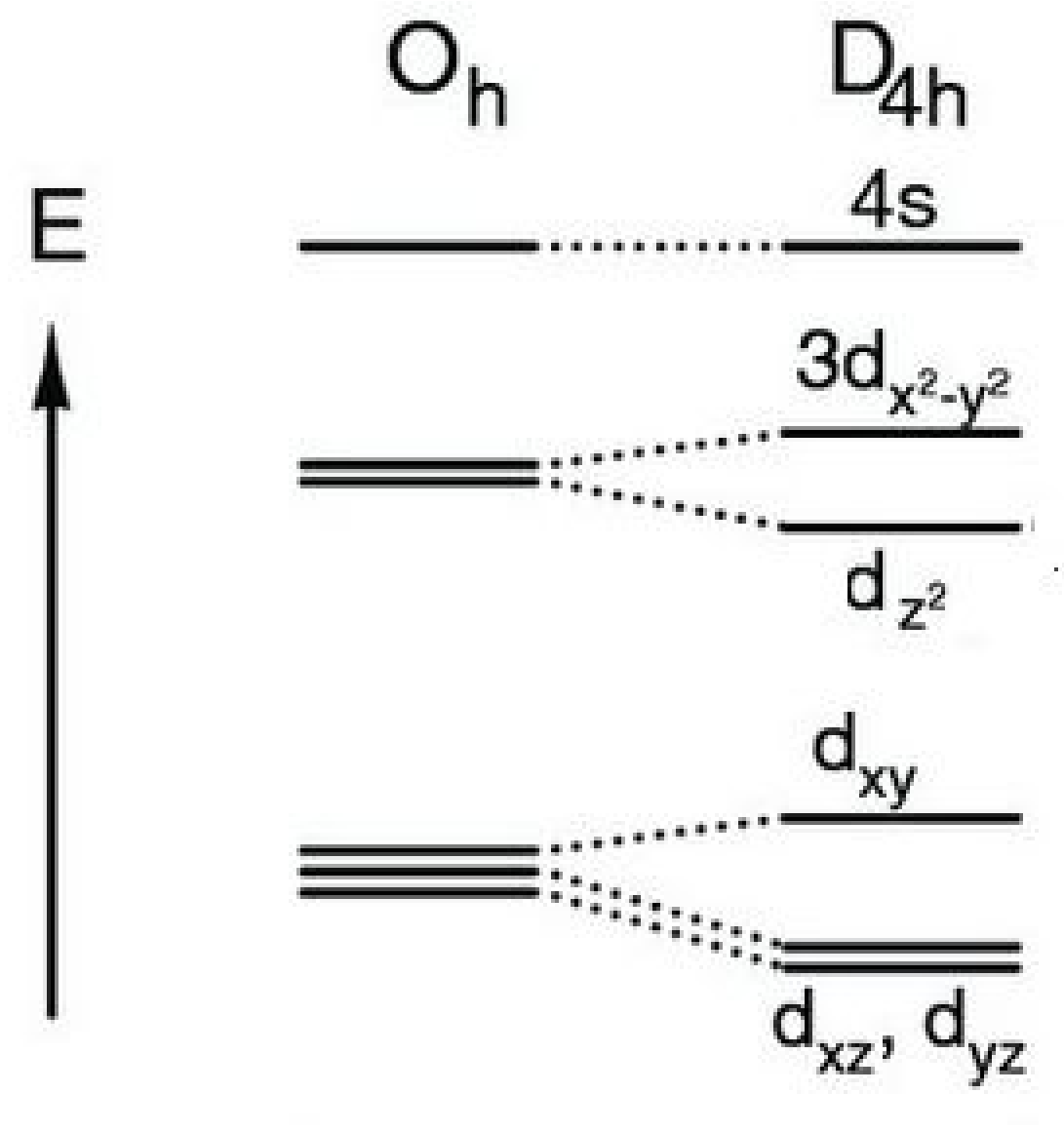


Scheme 1. Schematic representation of splitting of the 3d orbitals in $D_{4h}$ symmetry.

*Solid-state $^{13}C$ NMR spectrum*

Using the DFT-calculated isotropic NMR shifts, we constructed the NMR spectrum and compared calculated and experimental spectrum in Figure S11. Although the match between the measured and the calculated spectrum is not perfect, we see that the chemical shift range and the calculated shift values of the carbons are reasonably close to the experimental spectrum.

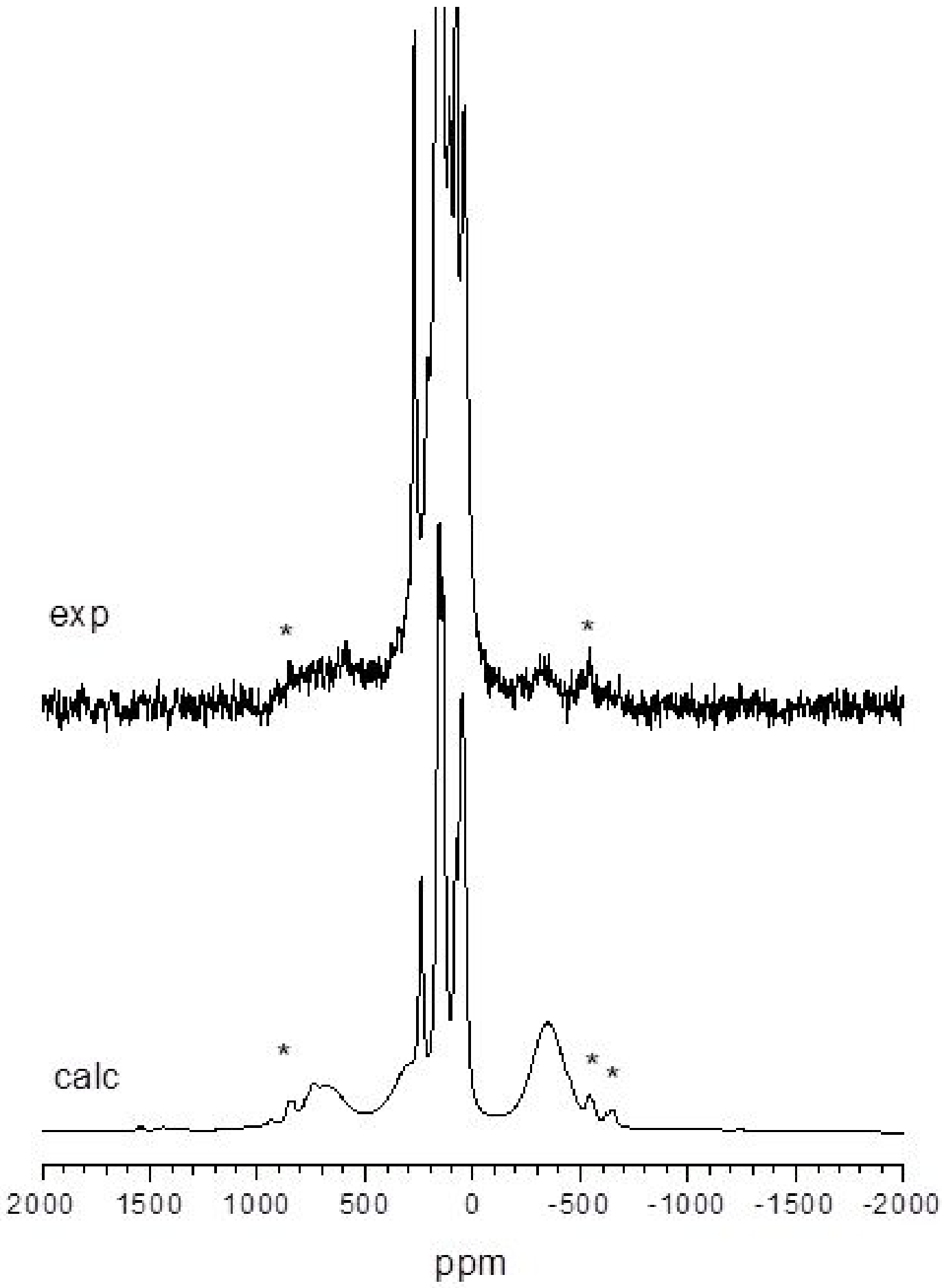


**Figure S11.** Comparison of the measured $^{13}C$ MAS NMR spectrum (exp) to the calculated one (calc). The asterisks denote the spinning sidebands.

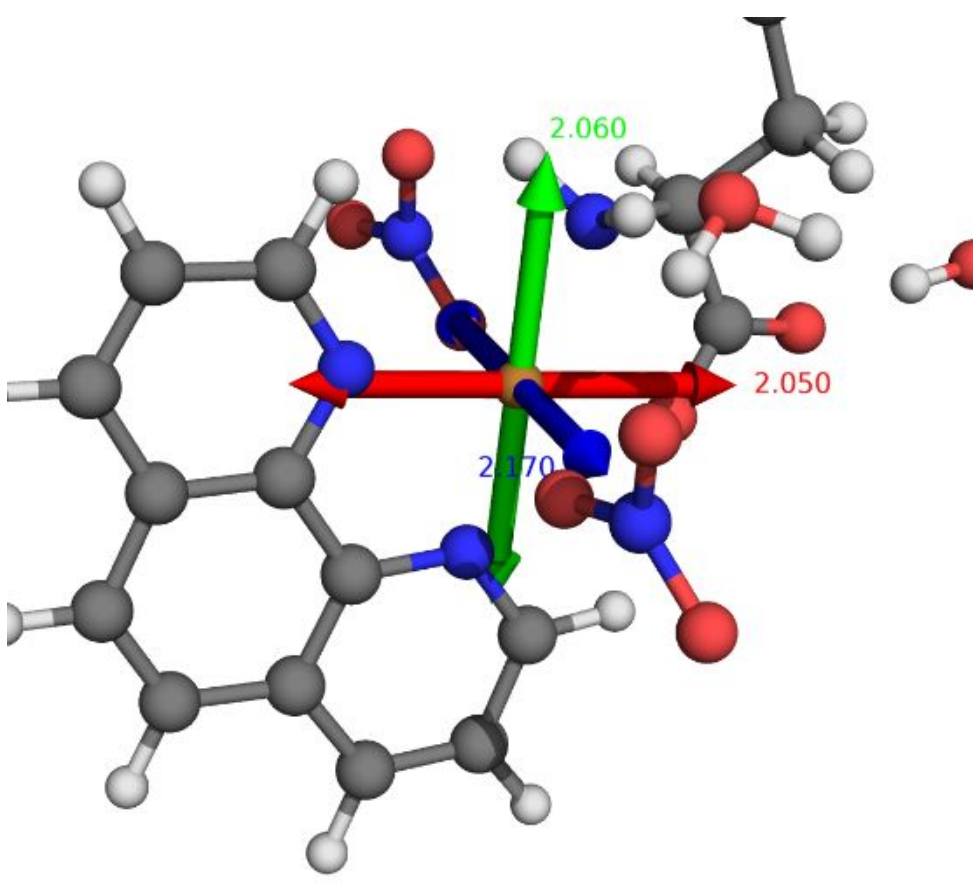


**Figure S12.** The principal components of the electronic **g**-tensor calculated using density-functional theory (SO-ZORA/PBE0/TZ2P).

**Table S6.** The parameters of the fitted peaks in the spectrum: *δ*(iso), the isotropic chemical shift in ppm; *δ*(CSA), the chemical shift anisotropy in ppm; *η*(CSA), the asymmetry of the chemical shift tensor; LB, the Lorenzian broadening in Hz; GB, the Gaussian broadening in Hz; the relative integral of the peak.

| SITES | 1 | 2 | 3 | 4 | 5 | 6 | 7 | 8 | 9 | 10 | 11 | 12 | 13 | 14 | 15 |
|---|---|---|---|---|---|---|---|---|---|---|---|---|---|---|---|
| δ(iso) | 156.5 | 272.7 | 214.8 | 39.0 | 73.7 | 80.4 | 120.9 | 154.6 | 104.7 | 194.3 | 749.0 | 569.9 | 339.0 | -316 | -518 |
| δ(CSA) | 300 | 350 | 350 | 350 | 350 | 350 | 350 | 350 | 350 | 350 | 350 | 350 | 350 | 350 | 350 |
| η(CSA) | 0.1 | 0.1 | 0.1 | 0.1 | 0.1 | 0.1 | 0.1 | 0.1 | 0.1 | 0.1 | 0.1 | 0,1 | 0.1 | 0.1 | 0.1 |
| LB | 827 | 714 | 661 | 1962 | 561 | 650 | 2302 | 515 | 990 | 2233 | 10 | 11 | 10 | 10 | 10 |
| GB | 0 | 0 | 300 | 0 | 0 | 0 | 2054 | 0 | 0 | 3139 | 9467 | 8378 | 8443 | 8689 | 5030 |
| Integral | 481 | 100 | 18 | 246 | 59 | 56 | 207 | 107 | 47 | 153 | 42 | 59 | 58 | 46 | 14 |

**Table S7**. The DFT-calculated $^{13}C$ NMR shifts of carbon atoms in **1** at 315 K.

| Atom | Hyperfine | Orbital | Total |
|---|---|---|---|
| C14 | -485.7 | 184.2 | -301.5 |
| C15 | -410.4 | 54.4 | -356.0 |
| C16 | 97.2 | 31.8 | 129.0 |
| C17 | 98.7 | 30.1 | 128.9 |
| C18 | -10.0 | 47.0 | 37.0 |
| C19 | -0.2 | 152.6 | 152.4 |
| C20 | -100.8 | 155.5 | 54.7 |
| C21 | 475.9 | 132.7 | 608.6 |
| C22 | -65.9 | 149.4 | 83.5 |
| C23 | 165.4 | 133.1 | 298.5 |
| C24 | 26.6 | 128.3 | 154.9 |
| C25 | 26.2 | 135.8 | 162.0 |
| C26 | 159.4 | 136.3 | 295.7 |
| C27 | -80.6 | 142.6 | 62.0 |
| C28 | 550.1 | 126.4 | 676.5 |
| C29 | 79.6 | 155.2 | 234.9 |
| C30 | -464.6 | 144.7 | -319.9 |
| C31 | -424.1 | 144.3 | -279.8 |
